# Optimized intranasal delivery of segesterone acetate progestin to the brain using nanoemulsions and microemulsions

**DOI:** 10.1007/s13346-025-01897-7

**Published:** 2025-06-18

**Authors:** Carina Peres, Sara Meirinho, Samille Ferreira da Silva, Izamara Maocha, Anabela Chiangalala, Susana Alves Ferreira, Márcio Rodrigues, Shimin Zhang, Narender Kumar, Regine Sitruk-Ware, Rui Caetano Oliveira, Carlos Gaspar, Ana Palmeira-de-Oliveira, Rita Palmeira-de-Oliveira, Gilberto Alves, Graça Baltazar, Adriana O. Santos

**Affiliations:** 1 RISE-Health, Faculty of Health Sciences, University of Beira Interior, Covilhã, 6200-506 Portugal; 2 BRIDGES - Biotechnology Research, Innovation and Design for Health Products, Polytechnic University of Guarda, Guarda, 6300-559 Portugal; 3 Population Council, Center for Biomedical Research, 1230 York Avenue, New York, NY 10065 USA; 4 Pathology Department, Centro Hospitalar e Universitário de Coimbra, Coimbra, Portugal; 5 Biophysics Institute, Faculty of Medicine, University of Coimbra, Coimbra, 3000- 548 Portugal; 6 Institute for Clinical and Biomedical Research (iCBR) Area of Environment Genetics and Oncobiology (CIMAGO), Faculty of Medicine, University of Coimbra, Coimbra, 3000-548 Portugal; 7 Labfit-HPRD, Health Products Research and Development Lda, Covilhã, 6200-284 Portugal

**Keywords:** Brain targeting, Intranasal, Microemulsion, Nanoemulsions, Safety, Segesterone acetate

## Abstract

Segesterone acetate (SA) is a selective and potent progesterone receptor agonist with potential application in the treatment of neurological diseases, such as multiple sclerosis and stroke. In this study, a microemulsion (ME) and three nanoemulsions (NEs) with a target SA concentration of 0.48 mg/g were developed for intranasal administration and extensively characterized regarding their physicochemical properties, stability, antimicrobial activity, and safety (in primary cortical cells, Hen’s Egg Test, MucilAir™ model and in rats). Plasma and brain SA levels were measured 30 and 60 min after intranasal administration in male rats.

The ME and the different NEs exhibited a droplet mean diameter of approximately 20 nm and 100 nm, respectively, with a low polydispersity index (≤ 0.1). Depending on their composition, they had neutral, positive, or negative zeta potentials and varying viscosities. The NEs exhibited good chemical and physical stability for 150–180 days at 4 °C, while the ME required a reduction in initial SA concentration to avoid drug precipitation. The NEs also demonstrated antimicrobial activity, although not ensuring effective antimicrobial preservation. The ME was the least cytotoxic of the formulations in vitro. No significant impairment of olfactory function or histopathological evidence of toxicity was observed in rats following single or repeated administrations of the ME and neutral NE at doses up to 40 µg/kg of SA. After a single intranasal administration of 40 µg/kg, SA brain concentrations exceeded 4 ng/g at 30 min, with no significant differences observed among the different formulation strategies. Notably, the ME led to a higher brain-to-plasma ratio (~ 7) at 30 min and thereby is a promising strategy to increase brain targeting.

**Graphical abstract:**

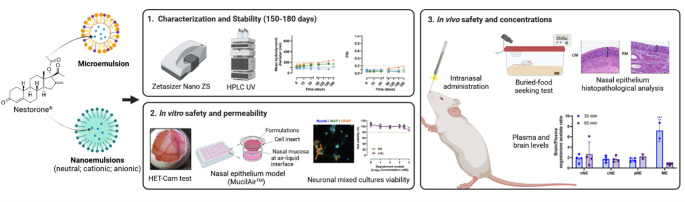

**Supplementary Information:**

The online version contains supplementary material available at 10.1007/s13346-025-01897-7.

## Introduction

Segesterone acetate (SA, also known as Nestorone^®^) is a potent and selective synthetic agonist of progesterone receptors [1]. Initially, SA was developed as a long-acting contraception methods in the form of silastic implants or intravaginal rings [2, 3]. In 2018, the Food and Drug Administration approved SA in combination with ethinyl estradiol as a contraceptive vaginal system under the brand name Annovera^®^ (NDA 209627 Food and Drug Administration, 2018). SA has no androgenic, estrogenic or glucocorticoid-like activities, and has recently been studied for the treatment of pulmonary inflammatory conditions [4], and as a promising neuroprotective agent in neurological diseases, such as multiple sclerosis, amyotrophic lateral sclerosis, spinal cord injury and stroke [4–10]. However, mostly due to its extensive hepatic first-pass metabolism, SA exhibits a limited oral bioavailability, which is a significant challenge considering its neuroactive potential [11–13]. In fact, pharmacodynamic studies on male and female rats demonstrated a potency 100-fold lower upon oral administration compared to subcutaneous administration [14]. While SA is not orally active, it is a very potent progestin when administered via non-oral routes [15]. Therefore, with the aim of achieving a more direct neuronal targeting of SA, there is a great interest in studying alternative administration strategies to deliver SA to the brain, such as the intranasal route of administration [6].

Intranasal drug delivery is a non-invasive approach that allows self-administration and fast drug absorption, with the potential for high bioavailability, particularly with low molecular weight drugs with lipophilic characteristics. The main reason lies in the highly vascularized (blood and lymphatic vessels) and permeable nasal epithelium for systemic absorption. The intranasal route also takes advantage of the direct anatomical connection between the nasal cavity and olfactory bulbs, allowing a direct diffusion or transport of drugs to the brain through the olfactory nerve pathway. Additionally, the nasal cavity is directly connected to medulla oblongata and pons cerebral regions by the trigeminal nerve, which may also contribute to the direct delivery of intranasally administered drugs [16, 17]. Nevertheless, bypassing the gastrointestinal tract and the first-pass hepatic metabolism is highly important to increase the bioavailability of drugs with high hepatic metabolization rate [18–20]. However, the small volume and the short residence time of preparations in the nasal cavity poses a challenge for nose-to-brain delivery of lipophilic drugs. This requires specific high strength formulation strategies that allow the administration of small volumes compatible with the nasal cavity capacity [18, 21]. In addition, the viscosity of the formulations must also be ideal to ensure that they are retained for long enough in the nasal mucosa, thus promoting the transportation of lipophilic drugs to the brain. A pH compatible with the nasal mucosa, preferentially in the range 5–6.5, and isotonicity or slight hypertonicity, are important critical quality attributes of aqueous nasal preparations. Besides it, the superiority of using carrier nanosystems (when compared to IN solutions) for brain delivery has been observed with a large set of drugs [19, 22].

Among the various nanosystems that can be explored, self-emulsifying drug delivery systems (SEDDS) and the related nano- and microemulsions, which form upon dispersion of SEDDS in aqueous environments (e.g., external aqueous phases, nasal mucus, gastrointestinal tract fluids), represent straightforward strategies for incorporating lipophilic drugs at high concentration [19]. These systems can enhance drug bioavailability across multiple administration routes, including the intranasal delivery [18, 23]. Depending on the qualitative and quantitative composition of the SEDDS and whether they form microemulsions (MEs) or nanoemulsions (NEs) after aqueous dispersion, these systems are classified as self-microemulsifying drug delivery systems (SMEDDS) or self-nanoemulsifying drug delivery systems, respectively [18]. The formed MEs and NEs, despite similar composition, have significant differences [24].

SMEDDS are mixtures of surfactants, organic solvents and lipids (in low proportion, usually < 20%) that, regardless of the sequence of components addition, easily and spontaneously disperse in water, forming isotropic and thermodynamically stable single-phase aqueous dispersions (MEs) [25]. Once formed, MEs are transparent or *quasi*-transparent because they consist of swollen micelles with nanometric sizes ranging from 10 to 100 nm, usually only slightly larger than the micelles formed by the surfactants. These micelles serve as carriers in which the lipid component and the lipophilic drug are dissolved. Conversely, NEs are thermodynamically unstable systems usually composed by a higher proportion of lipids that, when first mixed with surfactants and then dispersed in aqueous media, originate an opaquer liquid, with droplet mean size typically between 20 and 200 nm. Unlike MEs, NEs generally require high energy methods for formation [26]. Still, an innovative, simple NE vehicle compositions, which can contain different active substances, was previously prepared by our research team using an easy, low-energy mixture process, resulting in an extremely homogeneous NE with potential benefits in formulation’s performance and scalability [27]. Besides it, the team has also prepared a SMEDDS composed of Kolliphor^®^ RH 40, Transcutol^®^ and a lipid, showing to be highly successful regarding in vitro and in vivo efficacy and safety after intranasal administration [28–30]. Both the NE and the SMEDDS are very easy to prepare and adequate to solubilize high amounts of lipophilic drugs, maintaining a mean droplet diameter ≤ 100 nm and polydispersity index (PDI) < 0.1, which are important attributes for their successful translation. This justifies that these previous technologies were chosen as the basis for the development of novel SA intranasal formulations.

Thus, based on the described SMEDDS/ME and NE technologies previously developed by our team, the aim of the present work was to further develop different versions of NE and a ME of SA and assess them as safe intranasal SA formulations, able to originate potentially therapeutic SA brain drug levels. First, we optimized their composition to accomplish the ideal critical quality attributes aiming at future translation and a successful promotion of SA nose-to-brain delivery. Then, the most promising formulations were tested in vivo to confirm their safety and determine the plasma and brain concentrations achieved after their intranasal administration.

## Materials and methods

### Materials

Kolliphor^®^ RH 40 (Macrogolglycerol hydroxy stearate, a hydrophilic surfactant) and vitamin E acetate 98% (DL-alpha-tocopheryl acetate, an oil) were kindly offered by BASF (Ludwigshafen, Germany); Transcutol^®^ HP (Diethylene glycol monoethyl ether, a cosolvent), and Capryol^®^ 90 (Propylene Glycol Monocaprylate NF, an oil/hydrophobic surfactant) were kindly offered by Gattefossé (Saint-Priest, France); Imwitor^®^ 948 (Glyceryl Oleate, an oil/hydrophobic surfactant) was kindly offered by IOI Oleo GmbH (Hamburg, Germany). Ultra-pure water was obtained from a Milli-Q^®^ purification system from Millipore (Billerica, Massachusetts, United States of America). Polyethylene glycol 4000 (PEG) was acquired to Acofarma^®^ (Madrid, Spain). The cationic lipid cetalkonium chloride (benzyldimethylhexadecylammonium chloride hydrate, 97%) was acquired from Acros Organics B.V.B.A. (Geel, Belgium). SA (purity of 99.63%) was acquired either from Bld Pharmatech GmbH (Kaiserslautern, Germany) or Struchem Co., Ltd. (Wujiang, China). Acetonitrile and methanol used in high-performance liquid chromatography (HPLC)–- UV/DAD chromatography were HPLC grade, from PanReac AppliChem (ITW Reagents, Monza, Italy). Glycerin was from Labchem (Santo Antão do Tojal, Portugal). Carbopol 971P NF polymer was a gift from Lubrizol Advanced Materials Europe B.V.B.A. (Brussels, Belgium). Vicks Sinex Aloe, from Vicks Laboratory S.L. (Madrid, Spain) was bought at a local pharmacy and used as a control on in vitro safety experiments.

### Nanoemulsion preparation

The NE preconcentrate was first prepared by weighing and mixing excipients at an optimized predefined proportion [27]: Capryol^®^ 90 (50% w/w), Imwitor^®^ 948 (33.33% w/w) and Kolliphor^®^ RH 40 (16.67% w/w). To form the cationic NE (cNE), stock solutions of cationic lipid, i.e. cetalkonium chloride, in Capryol^®^ 90 were prepared at final concentrations of 1% or 2% (w/w). These solutions were then used instead of Capryol^®^ 90 to form cNE and 2cNE, respectively. After obtaining homogeneous preconcentrates, SA was dissolved at a target concentration of 22.4 mg/g (approximately its solubility limit in this preconcentrate).

As a first step, a concentrated NE was formed by mixing the preconcentrate and an aqueous phase at a proportion of 1:1. At this point, this aqueous phase was either water, a saline 20 mM phosphate buffered solution with different NaCl concentrations (as indicated in each case), a PEG 4000 solution (1% or 4% w: w), or a glycerol solution (2.6% w: w) [indicated in superscript in formulations code names on Tables S1 to S6 (Supplementary information)]. A low-energy phase inversion emulsification method was used: the total amount of the preconcentrate (with or without SA) was weighed; ¼ of the final aqueous phase mass was then weighed, added and manually mixed with the total amount of preconcentrate; finally, the remaining ¾ of the aqueous phase was further weighted, added and manually mixed to the previous mixture, forming the concentrated NE. Given the potency of SE, the target dosage strength was much lower than the obtained at this point. Therefore, an additional dilution step was taken to obtain the final NE at the target SA concentration (≤ 0.48 mg/g, equivalent to ≤ 2.1% preconcentrate) and to correct osmolality. The dilution was usually made in saline (e, g. NaCl 0.9%), or in an isotonic glycerol solution (2.6%) as a means to avoid interference with either the zeta potential of the cationic NE or with the viscosity of the dispersions of Carbopol. For the preparation of the anionic NE (aNE), a Carbopol dispersion was used at this final step. Carbopol was weighed and dispersed in water or a test isotonic aqueous phase at an initial concentration of 0.5%, the pH neutralized, and then further diluted to the intended concentration. The composition of the different batches prepared during this work is detailed in Supplementary information (Section S1, Tables S1, S3, S4 and S5, together with some of their attributes). For increased clarity, a summary of the selected formulations’ composition after an initial screening is given in Table 1.

**Table 1:** Summary of selected formulations’ composition

Code name	PC composition			Concentrated NE composition		Dilution of the concentrated NE/ME		
	Excipients	Mass ratio	Target [SA] (mg/g)	Components	Mass ratio	Target [SA] (mg/g)	Target [PC] (%)	Aq. phase
**nNE** ^**PEG 4%**^ **(nNE)**	Capryol^®^ 90: Imwitor^®^ 948: Kolliphor^®^ RH 40: cetalkonium chloride	3:2:1:0	22.4	PC: Aq. Solution of PEG 4000 at 4%	1:1	0.48	2.1	Aq. NaCl solution at 0.9%
**cNE** ^**Gly**^ **(cNE)**		2.97:2:1:0.03	22.4	PC: Aq. Glycerol solution at 2.6%	1:1	0.48	2.1	PC: Aq. solution of PEG 4000 at 4%
**aNE** ^**Peg 4%**^ **(aNE)**		3:2:1:0	22.4	PC: Aq. solution of PEG 4000 at 4%	1:1	0.48	2.1	PC: Aq. solution of PEG 4000 at 4%+ Carbopol at 0.1%
**ME**	Kolliphor^®^ RH 40: vitamin E acetate: Transcutol^®^ HP: water.	4:1:4:1	18.4	n.a.	n.a.	0.48	2.6	NaCl 0.9%

Aq., aqueous; ME, microemulsion; n.a., not applicable, NE, nanoemulsion; PEG, polyethylene glycol; PC, preconcentrate; SA, segesterone acetate

### Microemulsion preparation

The excipients composition of the SMEDDS was: Kolliphor^®^ RH 40 (40% w/w), vitamin E acetate (10% w/w), Transcutol^®^ HP (40% w/w) and ultra-pure water (10% w/w). The organic excipients were weighed and added to obtain a homogenous mixture, where SA was dissolved at a target concentration of 18.4 mg/g. Then, 10% of water was added and gently mixed to form the SMEDDS. Further dispersions using a saline solution were then performed to obtain oil-in-water (O/W) ME with SA target concentrations usually ≤ 0.48 mg/g (equivalent to ≤ 2.6% of preconcentrate) and correct osmolality to isotonic (or slightly hypertonic) values. The composition and respective attributes of the different batches prepared during this work are detailed in Supplementary information (Section S1, Tables S2 and S7). The composition of the selected ME is summarized in Table 1.

### Pharmaceutical characterization of the formulations

#### Dynamic and electrophoretic light scattering

Mean hydrodynamic diameter (droplet size) and PDI were measured by dynamic light scattering technique associated with cumulants method analysis using a Zetasizer Nano ZS Malvern^®^ (Malvern, United Kingdom) combined with the Zetasizer software (version 7.10). Zeta potential was measured in the same equipment by electrophoretic light scattering using the Dip Cell Kit. ME were directly measured without any additional dilution, while NE samples were diluted 25-fold using filtrated ultra-pure water before measurement. For each sample, three different measurements were automatically performed by the equipment set at 25 °C in two prepared independent discardable plastic cuvettes. When assessing the role of refrigeration in mean droplet size and PDI, formulations were placed at 4 °C at least overnight (or for predetermined times in the stability study), and then measurements were promptly performed exactly as stated before.

#### Osmolality measurements

Osmolality was measured in independent replicates using an Osmomat^®^ 3000 freezing point osmometer (Gonotec^®^ GmbH, Berlin, Germany). The device was previously calibrated using ultra-pure water and two standard solutions of 300 mOsmol/Kg and 850 mOsmol/Kg.

#### Rheological characterization

Viscosity measurements were performed in a Brookfield DV3T RVCP^®^ cone-plate rheometer (Brookfield Ametek, Massachusetts, USA) using the CPA-40Z cone spindle (viscosity measurement range of 1.7-32700 cP) and the Rheocalc T^®^ software (version 1.1.13). The temperature was maintained constant at 25 °C by a thermostatic water bath (MultiTemp III Thermostatic Circulator, Thermo Fisher Scientific, New Hampshire, USA). Equipment calibration was verified using Ametek Brookfield Fluid 500 Viscosity Standard (Middleborough, United States of America) with a standardized viscosity of 489 cP at 25 °C. An optimized formulation volume of 400 µL was placed on the plate, and the temperature was allowed to stabilize before measurements were taken.

For fluids with Newtonian rheological behavior, zero shear viscosity was determined at the highest torque value for a lower measurement error. For non-Newtonian fluids, zero shear viscosity was determined as the Y-intercept of the non-linear regressions based on the measurements performed at different shear rates at a constant temperature.

### Segesterone acetate assay by HPLC-UV-Vis/DAD

#### Apparatus and chromatographic conditions

Quantification of SA by HPLC was performed using an LC-2010 A HT Liquid Chromatography system coupled with an SPD-M20A diode-array detector, automatically controlled by the LabSolutions 5.52 data acquisition software, from Shimadzu (Kyoto, Japan). Chromatographic separation was performed at 30 °C on a reversed-phase column (C18, 55 × 4 mm, 3 μm particle size) protected by a reversed-phase pre-column (C18, 4 × 4 mm, 5 μm particle size), both LiChroCART^®^ Purospher^®^ STAR models (Merck, Darmstadt, Germany). Isocratic elution was carried out with a mobile phase containing a filtered and degassed mixture of methanol: water (50:50, v/v) pumped at a 1 mL/min. The sample injection volume was 20 µL and SA detection was performed at 241 nm in runs of 5 min.

#### Stock solutions, calibration standards, and quality controls

A stock solution of SA was prepared in methanol at a concentration of 1 mg/mL. From this solution, an intermediate solution at 10 µg/mL was also prepared by dilution in methanol. Different solutions for the calibration curve (0.05, 0.1, 0.498, 0.996, 2.491, 4.982, 9.963, and 24.908 µg/mL) and quality control (QC) samples (0.05, 0.149, 12.454 and 22.417 µg/mL) were prepared by diluting the stock or intermediate solutions in the mobile phase (methanol/water mixture 50:50, v/v).

#### Validation

Selectivity with respect to excipients of the formulation composition was determined by analyzing properly diluted blank samples of the different ME and NE in the mobile phase. Linearity was tested using calibration curves independently prepared on three different days, consisting of eight calibration standards covering a concentration range of 0.05–24.908 µg/mL, with 0.05 µg/mL being the lower limit of quantification (LLOQ). The obtained data was subjected to weighted linear regression analysis using different weighting factors, and the 1/x^2^ weighting factor was selected herein. Intraday and interday precision and accuracy were assessed by analyzing QC samples with SA concentrations representative of the calibration range [LLOQ (QC_LLOQ_) = 0.05 µg/mL, QC_1_ = 0.149 µg/mL, QC_2_ = 12.454 µg/mL, and QC_3_ = 22.417 µg/mL] independently prepared either in quintuplicate on the same day (*n = 5*), and in triplicate on three consecutive days (*n = 3*), respectively. Detailed results of all the studied parameters for this method validation are presented as Supplementary information (Section S2).

### Long-term stability evaluation

To assess chemical and physical stability, each formulation was sterilized by filtration (0.2 µM, regenerated cellulose, Ministart, Sartorius) and aliquoted into sterile glass vials to guarantee that microbial contamination would not interfere with the test. The baches were either characterized both before and after filter-sterilization or, in some cases, only after filtration. The aliquots were divided and stored at three different temperatures (4 °C, 25 °C, and 40 °C) for 180 days (batch 1) and 150 days (batch 2). At specified time points (0, 1, 7, 15, 30, 90, 150 or 180 days), the appearance was monitored, with particular attention to phase separation and precipitation of SA. In addition, the mean droplet size, PDI and zeta potential were also characterized as previously described. Simultaneously, the concentration of SA was determined using the validated HPLC assay described above (see the section “Segesterone acetate assay by HPLC-UV-Vis/DAD”).

### Evaluation of the antimicrobial effects of each formulation

#### Microorganisms and culture media

The microorganisms used in this study included the Gram-positive bacteria *Staphylococcus aureus* ATCC 6538, the Gram-negative bacteria *Pseudomonas aeruginosa* ATCC 9027 and *Escherichia coli* ATCC 8739, the yeast *Candida albicans* ATCC 10,231, and the filamentous fungus *Aspergillus brasiliensis* ATCC 16,404. The liquid culture medium were Mueller-Hinton (MH, VWR) for bacteria and RPMI-1640 (Biowest) for fungi and yeast, supplemented withMOPS (Fisher) 69.06 g/L, and D(+)-Glucose anhydrous (VWR) 20 g/L, at pH 7.0. The solid culture media included tryptic soy agar (TSA) for bacteria, sabouraud dextrose agar (SDA, VWR International, Carnaxide Portugal) for yeast, and potato dextrose agar (PDA; VWR, VWR International, Carnaxide Portugal) for filamentous fungus. All media were sterilized by autoclaving at 121 °C for 15 min before use.

#### Challenge test

To assess the efficacy of the formulations in preventing the growth of different microorganisms and, consequently, evaluate their preservative capacity without need for additional preservative agents, a challenge test was performed and interpreted according to the monograph 5.1.3 of the European Pharmacopoeia 10 [31]. For this purpose, 20 g of each formulation was inoculated with 200 µL of a yeast (*Candida albicans* ATCC 10231; 25 ± 2.5 °C, 2 days), a filamentous fungus (*Aspergillus brasiliensis* ATCC 16404; 25 ± 2.5 °C, 5 days), a Gram-negative bacteria (*Pseudomonas aeruginosa* ATCC 9027; 35 ± 2.5 °C, 18 to 24 h), and a Gram-positive bacteria (*Staphylococcus aureus* ATCC 6538; 35 ± 2.5 °C, 18 to 24 h). After inoculation, the mixtures were incubated for up to 14 days. At predefined time points post incubation (2, 7, and 14 days), the logarithmic reduction in microbial load of each microorganism was determined and compared with the validated acceptance criteria defined for inhalation preparations in the European Pharmacopoeia monograph. The residual antimicrobial activity of the formulations was neutralized by dilution in a validated neutralizing solution (buffered peptone water with polysorbate 80 at 3%, soy lecithin at 0.3%, saponins at 3%, and Triton X100 at0.1%, all from Prolabo, VWR International, Fontenay-sous-Bois, France) ensuring that it did not interfere with microbial recovery or the viability assessment of the microorganisms.

#### Determination of minimal inhibitory or lethal concentrations

The antimicrobial susceptibility testing followed the broth microdilution method, according to ISO 20776 standard [32] and the European Committee on Antimicrobial Susceptibility Testing (EUCAST) [33]. Briefly, the bacterial cultures were standardized to a 0.5 McFarland suspension using sterile 0.85% (w/v) NaCl and subsequently diluted in culture medium to achieve a final inoculum of 5 × 10^5^ CFU/mL Filter-sterilized formulations were subjected to duplicate serial dilution in 96-well microplates prior to the addition of microbial suspensions, resulting in an additional 2-fold dilution. The microplates included a positive control (growth control), which consisted of microbial suspensions inoculated in culture media, a negative control with non-inoculated culture media (to confirm the absence of contamination; and a sample sterility control where the sample was diluted in culture media (1:2) to assess sterility. The microplates were incubated at 37 °C during 18–- 20 h for bacteria, 24–- 48 h for yeast, and 74 h for molds. The minimum inhibitory concentration (MIC) was defined as the lowest concentration that caused a complete absence of visual microbial growth. To determine minimal lethal concentration (MLC), an aliquot from each well was transferred to agar to assess the lowest concentration at which no microbial growth was observed. Since the formulation also induced turbidity, an additional resazurin reduction assay was performed to confirm the visual inspection of MIC results. A 0.015% (w/V) resazurin solution in water was added (40 µL) to the plates, followed by incubation for 3–4 h. The presence of microorganisms was determined by colorimetric change, where wells containing viable microorganisms turned pink, while wells without microbial growth or viable cell remained blue.

### In vitro safety and segesterone permeation on human nasal tissue

MucilAir™ (Epithelix Sàrl, Geneva, Switzerland) was used as a commercial, three-dimensional and fully differentiated (air-liquid interface) in vitro model of the human nasal respiratory mucosa. It was obtained from a pool of healthy donors and supplied in inserts with an area of 0.33 cm^2^. Tissue inserts were maintained in an incubator at 37 °C with 5% CO_2_ and treated according to manufacturer’s instructions and recommendations. MucilAir™ medium was renewed every 2 or 3 days. Every 3 weeks, mucus was washed from the apical side of the inserts with Krebs-Ringer Bicarbonate buffer (KRB, NaH_2_PO_4_∙H_2_O 1.5 mM, Na_2_HPO_4_ 0.83 mM, KCl 4.56 mM, NaCl 119.78 mM, MgCl_2_∙6H_2_O 1.67 mM, NaHCO_3_ 15 mM, CaCl_2_∙2H_2_O 1.2 mM, and D-glucose 10 mM, pH 7.2), previously equilibrated in the incubator at 37 °C with 5% CO_2_. For that, 200 µL of buffer was added to each insert and incubated for 20 min. The buffer was then carefully removed and the plate was returned to the incubator.

The in vitro safety of the formulations on human nasal tissue was assessed in MucilAir™. For that, the transepithelial electrical resistance (TEER) was measured to assess the integrity of the epithelial barrier, the resazurin reduction assay was performed to assess cellular metabolism/cell viability, and the semi-quantification of lactate dehydrogenase (LDH) release to the basal medium was done to study cell death/lysis.

Mucous removal and tissue resistance readings for TEER calculation were performed three days before the assay. On the day of the assay, an independent 24-well plate containing only 400 µL of culture medium was placed in the incubator to equilibrate at 37 °C with 5% CO_2_ for approximately 15 min. On MucilAir™ 24-well plates, treatments were performed by adding 16.5 µL of each formulation to be tested or control solutions (0.9% NaCl, 0.2% benzalkonium chloride, Vicks^®^ as reference product control, and 1% Triton X-100) to the apical side of the inserts (equivalent to 50 µL/cm^2^). Then, the plate was incubated for 30 min under the previously described conditions. Subsequently, the inserts were transferred to a new 24-well plate containing pre-heated KRB buffer and the remaining medium of the basal side was stored at 4 °C for further lactate dehydrogenase (LDH) and SA quantification. Then, 200 µL of the equilibrated KRB buffer were added to the apical site for reading the tissues’ resistance. Finally, the KRB buffer was removed, and the inserts were transferred to a new plate containing 400 µL of resazurin solution prepared on equilibrated KRB buffer (30 µM), with 200 µL of the same resazurin solution also being added to the apical side. The plate was then incubated for 1 h. At the end of the incubation, 180 µL of the blank resazurin solution and of the resazurin solution incubated on each apical insert side were transferred to a 96-well plate for the resazurin reduction quantification. The inserts were then transferred to wells with KRB, washed, and then transferred and incubated with fresh culture medium.

#### Lactate dehydrogenase semi-quantification

Fifty µL of the culture medium removed from the basal side of each insert were added to a low UV absorption 96-well plate (UV-STAR^®^, Greiner Bio-One) (*n* = 3). In parallel, the basal medium from the positive control well lysed with 1% Triton X-100 was diluted in blank medium to plot a semi-quantitative calibration curve of different relative LDH concentrations (0–100% LDH) and then added to the same plate (50 µL/well). To each of these wells containing medium, 200 µL of NADH (253 mM) were added, followed by 25 µL of pyruvate (15 mM). Finally, absorbance was measured at 340 nm using a plate spectrophotometer (Bio-Rad Laboratories, Hercules, USA) after 3 h of pyruvate addition. The reduction in NADH absorption was calculated as the difference between fresh medium well (0% LDH) and test/reference medium well, with the results being interpreted using the equation obtained from the LDH semi-quantitative calibration curve. Linearity was achieved between concentrations of 0% and 25%. Finally, normalized LDH release was calculated by subtracting the LDH quantified on negative controls (NaCl 0.9% wells).

#### Transepithelial electrical resistance

For tissue resistance measurements, an Epithelial Volt/Ohm Meter (EVOM™) from World Precision Instruments (Sarasota, Florida, USA) was used. KRB was placed in a Petri dish and incubated at 37 °C with 5% CO_2_ for approximately 15 min to reach equilibrium. The EVOM™ electrode was decontaminated in a Falcon tube containing 70% ethanol, then placed in sterile KRB at room temperature for about 2 to 3 min. The volume of KRB should always be less than that of the ethanol used for electrode sterilization. For the measurement, 200 µL of pre-equilibrated KRB from the incubator was added to the apical side of the inserts, followed by the insertion of the EVOM™ electrode and recording of the measured value. Subsequently, the electrode was decontaminated again, the KRB previously placed on the apical side of the inserts was carefully removed without damaging the tissue, using forceps to lift and tilt the insert, and the plate was placed back in the incubator. The tissues’ TEER value (Ω·cm²) was calculated using the following equation (Eq. 1):

1 $$\:TEER\:=\:(Measured\:resistance\:\Omega\:-\:100\:\Omega\:)\:\times\:\:0.33\:cm^2\:$$

where 100 Ω represents the resistance of the insert without tissue, and 0.33 cm^2^ is the area of the insert.

#### Resazurin reduction assay

Reduced resazurin fluorescence measurement was performed from the top of a 96-well plate on a spectrophotometer (SpectraMax Gemini EM, Molecular Devices, San Jose, CA) at excitation and emission wavelengths of 544 and 590 nm, respectively (automatic cut-off). The percentage viability was calculated in comparison with the mean fluorescence of the negative control wells (cell culture medium plus resazurin).

### In vitro irritancy evaluation by hen’s egg test- chorioallantoic membrane

Aiming to evaluate the in vitro irritancy potential of different formulations, the Hen’s Egg Test–- Chorioallantoic Membrane (HET-CAM) test was performed herein. This test consists in evaluating irritancy in the chorioallantoic membrane of a chicken egg through observation of macroscopic alterations in vasculature (hemorrhage, coagulation and/or vessel lysis). The test was performed according to the ICCVAM-Recommended Test Method Protocol “HET-CAM Test Method” [34]. Fresh fertile White Leghorn chicken eggs weighing an average of 45–65 g were purchased to Granja Santa Isabel (Spain) aviary. Before being used, eggs were maintained on an automatic rotating tray at a temperature of 37.8 °C ± 0.3 °C and a relative humidity of 58% ± 2 (Brinsea Ovation 56 EX) for 9 days, being inspected on the 8th day to check for the presence of an embryo. The incubation was then continued for one more day, keeping the eggs immobilized for stabilization of the chorioallantoic membrane. On the 9th day, the chorioallantoic membranes were exposed by removing the eggs’ shell, always checking the membranes for the eventual observation of lesions. Then, 0.3 mL of the test substance were directly added (without dilution) to its surface (*n = 3*). The time (in minutes) for the observation of hemorrhage, coagulation and/or vessel lysis was then recorded up to 5 min. Each of these assessments were individually considered (Table 2) and then combined to derive a score which is used to classify the irritancy level of the test substance. If the score is ≤ 9, the substance is considered non-severely irritating, and if it is > 9, the substance is considered severely irritating.

**Table 2:** Scoring criteria for the HET-CAM test method

Reactions	Scoring		
	0.5 min	2 min	5 min
Vascular lysis	5	3	1
Hemorrhage	7	5	3
Coagulation	9	7	5

Positive controls were an aqueous solution of sodium hydroxide (NaOH, VWR) at 0.1 N, and an aqueous solution of sodium dodecyl sulfate (SDS, Thermo Scientific) at 1% (w/v); the negative control was an aqueous solution of NaCl at 0.9% (w/v) (Merck). Representative photographs were taken of the chorioallantoic membrane of one egg of each test group, before beginning the test and at the end of the test.

### In vitro safety assessment on primary cultures of cortical cells

#### Preparation of primary cultures of cortical cells

To obtain embryos at 15 to 16 days of development, pregnant Wistar rats from local certified facilities (Health Sciences Research Center, University of Beira Interior) were euthanized under deep anesthesia with 5% isoflurane. Subsequently, the abdominal cavity was opened, and the embryos were removed and placed in a tube with 50 mL of phosphate-buffered saline (PBS) at 4 °C. On average, 1 to 2 pregnant females and 7 to 11 embryos were used for each independent cellular preparation.

Immediately after removal, embryos were transported to the culture room for cell isolation and culture. There, brains were separated, meninges were removed, and the cortices were dissected and transferred to a tube with Hanks’ balanced salt solution (HBSS) solution without Ca^2+^ and Mg^2+^ (8.006 g/L NaCl; 0.3996 g/L KCl; 0.35 g/L NaHCO_3_; 0.0604 g/L Na_2_HPO_4_; 0.042 g/L KH_2_PO_4_; 0.09 g/L D-glucose; 0.012 g/L sodium pyruvate; 2.383 g/L HEPES, final pH 7.2). Following this procedure, enzymatic dissociation was performed for 10 min at 37 °C in a solution with 2 mg/mL trypsin and 0.5 mg/mL DNase. After this period, 10 mL of a 10% fetal bovine serum (FBS) solution was added to stop the trypsin action, the tissue was centrifuged at 140 *g* for 1 min, and the obtained cell suspension was filtered and subsequently centrifuged at 300 *g* for 3 min. The supernatant containing some cellular debris was discarded, and the cell sediment was resuspended in Neurobasal™ Medium (NBM, Gibco, Cat: 21103-049) heated to 37 °C and supplemented with 0.5 mM glutamine (Sigma-Aldrich, Cat: G3126), 0.12 mM gentamicin (Sigma-Aldrich, Cat: G1272), 2% B27 (Gibco, Cat 17504044), and 10% FBS (Biochrom AG, Cat: S0115). The total number of cells in the cell suspension was determined by counting in a Neubauer chamber.

To obtain mixed cortical cultures, cells were cultured at a density of 0.2 × 10^6^ cells/cm^2^ in NBM supplemented as described above. A volume of 0.25 mL/well was added to 48-well plates, previously coated with poly-D-lysine (Sigma-Aldrich, Cat: P1024). The conditions used for these cultures (media and cell density) were previously defined in earlier studies [35]. Cells were then maintained in culture in an incubator under defined environmental conditions (humidity, 5% CO_2_ at 37 °C) until the 11th day of culture, at which point experimental procedures were initiated.

#### Cell viability assay

The viability of cortical cells was assessed by a metabolic activity assay 24 h after cells’ contact with the nNE and ME at different final concentrations of SA (1–10000 nM). Control cells were incubated with each formulation without SA diluted in DMSO. Twenty-four hours after formulations’ incubation, the cell culture medium was removed and a 3-(4,5-dimethylthiazol-2-yl)-2,5-diphenyltetrazolium bromide (MTT) solution in HBSS (0.5 mg/mL) was incubated for 1 h at 37 °C. Subsequently, the MTT solution was also removed, and the blue formazan crystals were dissolved with acidified isopropanol (0.04 M HCl in isopropanol). Absorbance was read (xMark™ Microplate Spectrophotometer, Bio-Rad) at 570 nm using a reference filter at 620 nm. Cell viability was expressed as a percentage of the mean absorbance of control wells. The data presented represent the mean of least 3 three independent cellular preparations, with each experimental condition being performed in triplicate.

### Animals

Adult male Wistar Han rats, aged between 10 and 11 weeks and weighing from 240 to 324 g, were obtained from local certified facilities (Health Sciences Research Center, University of Beira Interior). Animals were housed under controlled environmental conditions (12/12-hour light/dark cycles, 20 ± 2 °C temperature, and 50 ± 5% relative humidity), receiving standard 4RF21 rodent food (Mucedola, Italy) and sterile tap water *ad libitum*. The experimental protocols were reviewed and approved (reference 0421/000/000/2023 002871) by the competent national authority [Portuguese General Directorate for Food and Veterinary (DGAV – Direção Geral de Alimentação e Veterinária)], in agreement with the regulations of the European Directive 2010/63/EU and Portuguese law (*Decreto-Lei nº 113/2013*).

### In vivo safety evaluation

For in vivo safety evaluation, male rats were divided in 10 independent experimental groups, with 4 animals/ group:

- Group 1 and 2: negative toxicity control groups (single or repeated intranasal administration of NaCl 0.9%, respectively).
- Group 3 and 4: positive toxicity control groups (single intranasal administration of ZnSO_4_ 10% or repeated intranasal administration of NaCl 0.9% followed by one intranasal administration of ZnSO_4_ 10%).
- Group 5 and 6: intranasal administration with nNE containing SA (single dose or repeated doses of 10 µg/kg, respectively).
- Group 7 and 8: intranasal administration with nNE containing SA (single dose or repeated doses of 40 µg/kg, respectively).
- Groups 9 and 10: intranasal administration with ME containing SA (single dose or repeated doses of 40 µg/kg, respectively).

These doses of 10 and 40 µg/kg were selected in the range of previously used SA doses in experimental stroke models (10–80 µg/kg [6, 9]), and for allowing an adequate volume for intranasal administration (maximum around 25 µL per animal of 300 g for formulations at the target concentration of 0.48 µg/mL SA). The groups that only received a single intranasal dose were administered 6 h after being subjected to food deprivation (maintaining access to water *ad libitum*). As for the groups that were subjected to repeated dose regimens, the animals received a daily intranasal administration during 7 consecutive days, with the last two doses being halved to avoid possible side effects caused by abrupt SA withdrawal [9]. Before the beginning of the administrations, SA concentration of each formulation was determined by HPLC (see the section “Segesterone acetate assay by HPLC-UV-Vis/DAD”).

The intranasal administration of formulations/solutions were performed after anesthesia with a 3% (v/v) isoflurane rate combined with 1000 mL/min of oxygen. Rats were placed in a supine position with their head tilted back, and the formulations were administered using a 50 µL Hamilton syringe (Nevada, United States of America) coupled with a Braun Introcan^®^-W Certo (19 mm) flexible catheter (Melsungen, Germany). The catheter was inserted a few millimeters through each nostril and the volume was divided between the two sides of the nasal cavity. The administration occurred rapidly, within approximately 30 s after induction of anesthesia, and each administered animal was held in supine position for a few more seconds until it awakened from the anesthesia.

#### Olfactory toxicity and body weight evaluation

Buried food seeking test takes advantage of the highly developed olfactory sense of rodents for foraging, orientation, navigation and other natural activities [28]. Therefore, this test was used to assess if the formulations under test and the different SA doses could cause damage to the olfactory mucosa and, consequently, olfactory impairment on animals subjected to single or repeated administration. For that, 24 h after animals being subjected to food deprivation (18 h after the single administration in the single dose groups, and 24 h after the last administration of a 7-day administration regimen in the repetitive dose groups), starved rats were individually placed in clean cages containing 8 cm-thick corn-cob bedding, being allowed to explore it for around 30 min. Then, rats were removed, and a 3 g chow pellet was randomly and deeply buried under the bedding surface. After, each rat was placed back into the cage, and the time between the moment that was placed (t0) and the time at which the animal undercovers and starts to eat the pellet was scored as the latency to find the food (measured in seconds, up to a maximum of 300 s). All experiments were video-recorded for further analysis.

Besides the analysis of olfactory sense, the animals assigned to the repeated dose groups were individually weighed right before each intranasal administration. This allowed for the assessment of body weight changes over the 7 days of administration, with weight loss being interpreted as a possible sign of toxicity.

After completing the tests on the groups subjected to repeated dose regimens, rats were euthanized under deep anesthesia [5% (v/v) isoflurane] by cervical decapitation. Then, nasal cavities were carefully excised, fixed with 10% formaldehyde solution for 72 h, and subsequently processed for histological evaluation of the nasal mucosa.

#### Histological evaluation

After fixation, nasal cavities were decalcified with Decalcifier II (Surgipath, Leica Biosystems, IL, USA) until complete decalcification, followed by dehydration with an increasing gradient of ethanol, xylene, and then embedded in paraffin. Paraffin blocks were sectioned using a microtome, with 5 μm thickness, and then stained with Hematoxylin and Eosin (H&E), and periodic acid–Schiff (PAS). Finally, the histopathological evaluation of potential toxicity signs in both olfactory and respiratory mucosa was performed by a trained pathologist under an optical microscope.

### Plasma and brain levels of segesterone acetate

To understand which of the developed formulations were the most promising ones to promote SA brain targeting after a single intranasal administration, 32 rats were randomly divided in 4 independent experimental groups, each receiving a different intranasal formulation (ME; nNE, aNE, or cNE) at a SA dose of 40 µg/kg. As mentioned in the previous section, this dose was selected in the range of previously used SA doses in experimental stroke models (10–80 µg/kg), and for allowing an adequate volume for intranasal administration.

After anesthesia, an intranasal formulation volume between 19.7 µL and 26.9 µL was administered divided between the 2 sides of the nasal cavity (see the section In vivo safety evaluation). At two predetermined time-points after SA administration (30 and 60 min; *n* = 4 per time-point), rats were again subjected to deep anesthesia, blood was collected by tail puncture to EDTA tubes and euthanized by cervical decapitation. Brains were immediately harvested and gently washed with NaCl 0.9% to remove most of the remaining blood. Blood samples were centrifuged at 3351 g for 10 min at 4 °C to obtain plasma samples. Plasma and brains were stored at − 80 °C until further processing. Then, frozen brains were cut longitudinally, and half of the frozen rat brain was weighted, and the other half was kept as a reserve sample. A cold mixture of water with 5% methanol was added at the proportion of 4 mL per gram of tissue, followed by tissue homogenization with an Ultra-Turrax^®^, on ice, at 12 rpm, for 3 cycles of 30 s. The Ultra-Turrax^®^ blade was thoroughly washed with clean water (2 times), methanol, and homogenization solution between samples. Homogenates were aliquoted and stored at − 80 °C until analysis.

#### SA bioassay

SA concentrations in plasma and brain homogenates were measured using a validated LC-MS/MS method at Population Council, NY.

The SA method validation was performed by using eight calibration standards in the range between 0.05 and 10 ng/mL for SA in plasma, and seven calibration standards in the range between 0.1 and 10 ng/mL for SA in brain homogenates. A labeled internal standard (IS) was used for normalization purposes. Three separate quality control samples were also prepared by spiking the matrix (rat plasma / brain homogenates) at 0.15, 0.8 and 8 ng/mL concentrations. Specificity, linearity, LLOQ, accuracy, precision, matrix interference effects, recovery and stability were evaluated and validated prior to sample analysis. Each sample analysis run included at least 7 levels of calibration standards, one matrix blank, one matrix blank spiked with internal standard, and three levels of quality control samples (*n* = 2). One set of quality control was placed in front of the sample queue and another set at the end of the sample queue.

To extract SA from samples, 50 µL of plasma or brain homogenate were mixed with 100 µL methanol containing IS (2.5 ng/mL) and vortexed for 20 min. The samples were then centrifuged at 14.5 × 10^3^ rpm for 10 min (Eppendorf, Minispin) and 100 µL supernatant from each tube was transferred into HPLC vials and 5 µL injected into the chromatographic system. A Waters Acquity Premier system was used for setting the reverse-phase LC conditions. The separation of SA was performed on Waters Acquity Premier BEH C18 1.7 μm VanGuard FIT 2.1 × 50 mm column by gradient elution using the mobile phase A (5 mM ammonium formate and 0.1% formic acetic in water containing 10% methanol) from 50 to 20%, and the mobile phase B (methanol) from 50 to 80% over 4 min, at a flow rate of 0.25 mL/min to elute SA and IS.

The SA was quantified by positive electrospray ionization in multiple reaction monitoring (MRM) mode using the Waters Xevo TQ- Absolute mass spectrometer system (Waters, Milford, MA). Quantification was performed by monitoring the transition from 371.1289 m/z to 253.0953 m/z for SA and from 374.1727 to 253.0863 m/z for IS (13C3 SA). MassLynx software version 4.2 was used to control all parameters of the LC-MS/MS system. The SA calibration curves were linear, and LLOQ for rat serum and brain homogenates were 50 pg/mL and 100 pg/mL, respectively. The intra- and inter-assay coefficients of variations were < 15% for both serum samples and brain homogenates.

### Statistical analysis

Graphics and statistical data analysis were performed using GraphPad Prism^®^ software, version 10.0.2. Data were presented as mean ± standard deviation (SD). The SA permeation through MucilAir™ was analyzed by one-way ANOVA followed by Tukey’s post-hoc test. The results obtained in the buried food seeking test were analyzed with two-way ANOVA followed by a Dunnet’s post-hoc test. In vivo brain/plasma ratios were calculated by dividing the SA concentrations obtained in the brain by the respective concentrations obtained in the plasma and then analyzed by a two-way ANOVA using Sidak’s post-hoc test. Differences were considered statistically significant when the *p*-values were less than 0.05 (* *p* < 0.05, ** *p* < 0.01, *** *p* < 0.001, and **** *p* < 0.0001). To the viscosity vs. shear rate data, a two-phase decay nonlinear regression analysis was applied to determine the zero-shear viscosity of non-Newtonian fluids.

## Results

### Development and stability of segesterone acetate formulations

Several new isotonic or hypertonic SA-NE and SA-ME compositions were screened by varying the preconcentrate and the aqueous phase of previous versions of NE and SMEDDS vehicles [27]. The droplet size and other physical attributes of the compositions and the respective changes throughout experiments are described in Supplementary information (Section S1). From the initial trials, an isotonic neutral NE (**nNE**^**PEG 4**%^, for simplification named **nNE**), a cationic NE (**cNE**), and an anionic and viscous NE (**aNE**^**PEG 4%**^, with Carbopol at 0.1%, for simplification named **aNE** from now on) were selected for further comparison alongside a ME formulation.

**Fig. 1:**
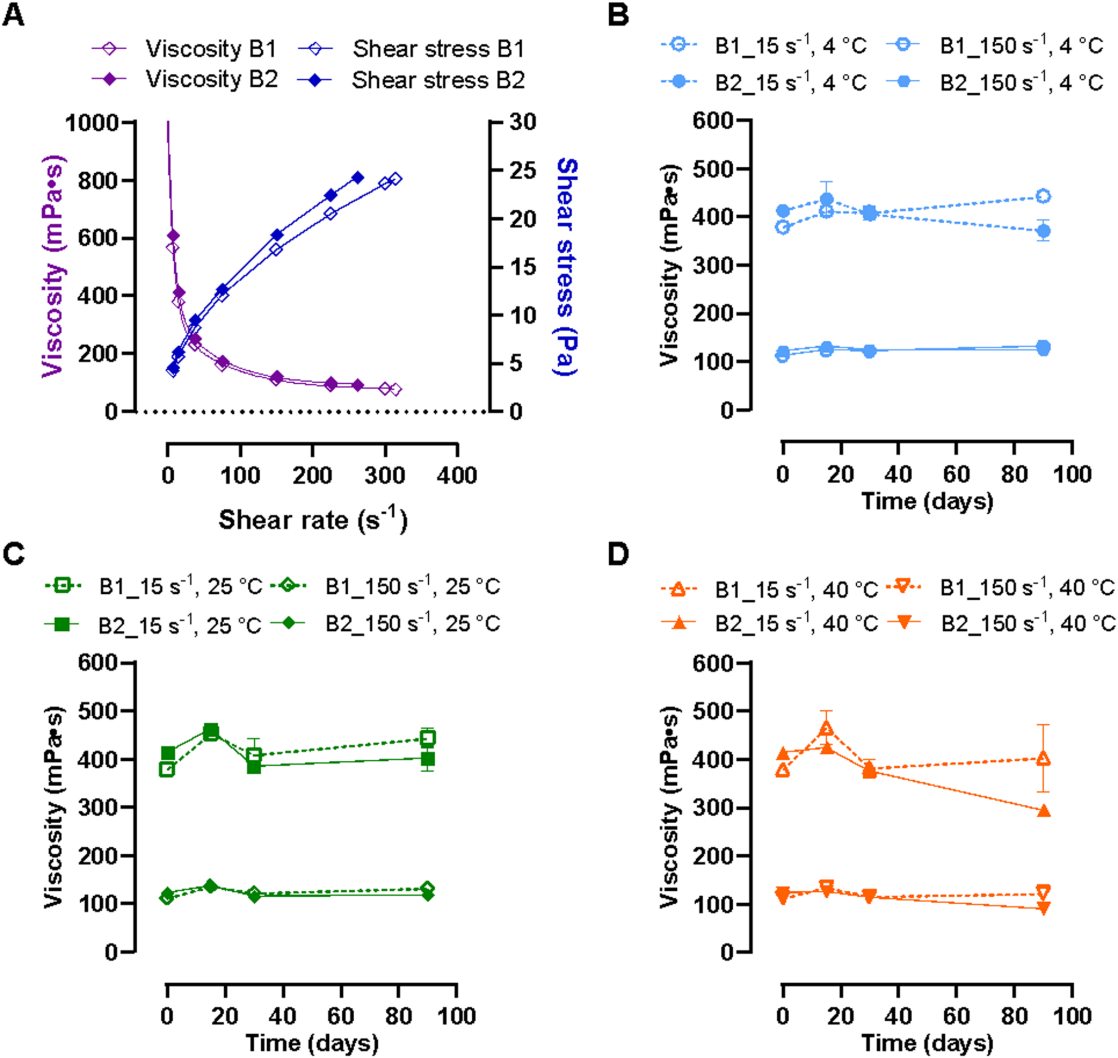
Rheological characterization of two batches of the selected anionic and viscous segesterone acetate nanoemulsion (aNE). Viscosity and shear stress at varying shear rates is shown for the day of formulation preparation (**A**) and viscosity of formulations stored at 4 °C (**B**), 25 °C (**C**) and 40 °C (**D**) for 90 days is displayed for 2 example shear rates over time. All measurements were performed at 25 °C. Each batch was measured twice and data correspond to mean ± standard deviation

The ME, the nNE, and the cNE had low viscosity, as they were highly diluted emulsions in simple aqueous solutions. In contrast, the aNE obtained with addition of Carbopol to the aqueous phase at 0.1% had increased viscosity and a non-Newtonian reofluidifying behavior (Fig. 1A). Its rheological characteristics were evaluated over time in two different batches stored at different temperatures (4 °C, 25 °C and 40 °C, Fig. S1) and maintained this behavior during the 90 days of study, with no influence of storage temperature on the rheological characteristics of the formulation. As it can be observed in Fig. 1B and D, viscosity values measured at shear rates of 15 s^− 1^ and 150 s^− 1^ were close to 400 and 100 mPa·s, respectively. These values did not vary significantly over the 90 days of storage at different temperatures.

**Fig. 2:**
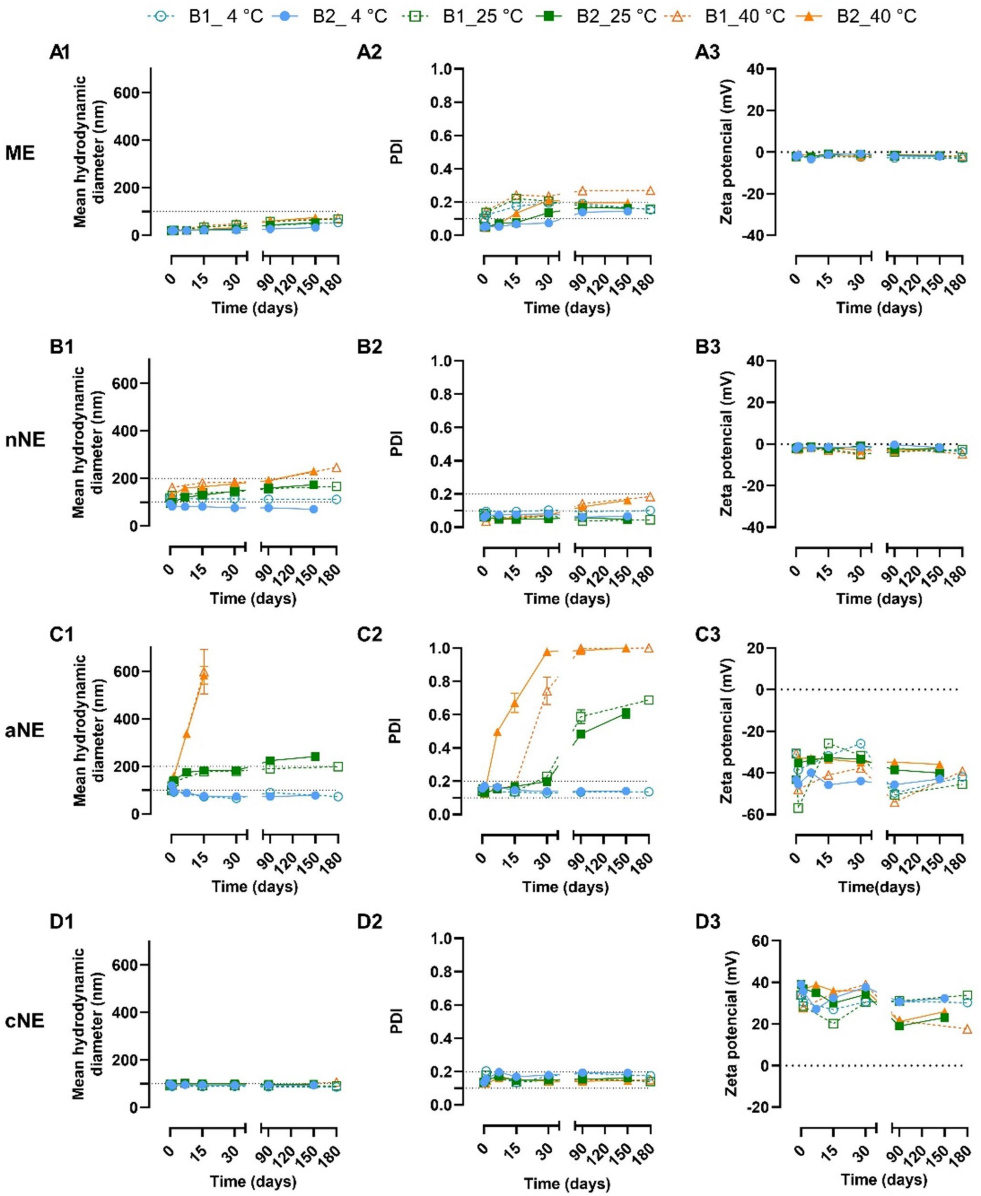
Droplet mean size, polydispersity index (PDI) and zeta potential of the selected formulations over time: a microemulsion (ME, **A1**– **A3**), a neutral nanoemulsion (nNE, **B1**– **B3**), an anionic nanoemulsion (aNE, **C1**– **C3**) and a cationic nanoemulsion (cNE, **D1**– **D3**). Two different batches (**B1** and **B2**) were evaluated over 150 or 180 days after preparation and data correspond to mean ± standard deviation of 2 or 3 independent sample dilutions of each formulation. Mean hydrodynamic diameter data of the aNE stored at 40 °C is absent from day 30-on because it lacked quality due to high heterogeneity

The mean droplet size, PDI, and zeta potential, as well as physical and chemical stability of two independent batches of the selected formulations was evaluated up to 6 months at different storage temperatures. Filter-sterilized formulations were used, and it was confirmed that filtration did not alter formulations’ attributes (examples of that are given in Supplementary information, Tables S3, S4, S5 and S7). Regarding the mean droplet size of the ME, it was initially 19 nm and increased over time up to the 180 days of testing, an increase of about 2-fold, less marked when ME was stored at 4 °C (Fig. 2A1); nevertheless, mean size never reached the cut-off value of 100 nm. Over time, the ME droplet size also became more heterogeneous, particularly in batch nr 1 (B1) and when ME were stored at 40 °C (Fig. 2A2). ME’s zeta potential was neutral and remained stable throughout the study period, as expected (Fig. 2A3).

The nNE, cNE and aNE displayed stable mean droplet size, PDI, and zeta potential for the entire study duration when stored at 4 °C (Fig. 2, B1 to D3). However, only the cNE was stable at 25 °C (and even 40 °C) in terms of all three studied parameters (Fig. 2, D1 to D3). In fact, it was evident that size increased right after the first day of preparation when both the nNE and aNE were stored at 25 °C and 40 °C. Nevertheless, the nNE stored at 25 °C had a stable PDI < 0.1 up to the 150th or 180th day after preparation, and the mean droplet remained below 200 nm for at least 6 months. As for the aNE stored at 25 °C, it showed a marked PDI increase 30 days after preparation. In general, the zeta potential of all the formulations stored at the three temperatures studied remained stable over the entire study, with slight fluctuations in charge values only occurring in the aNE and the cNE.

**Fig. 3:**
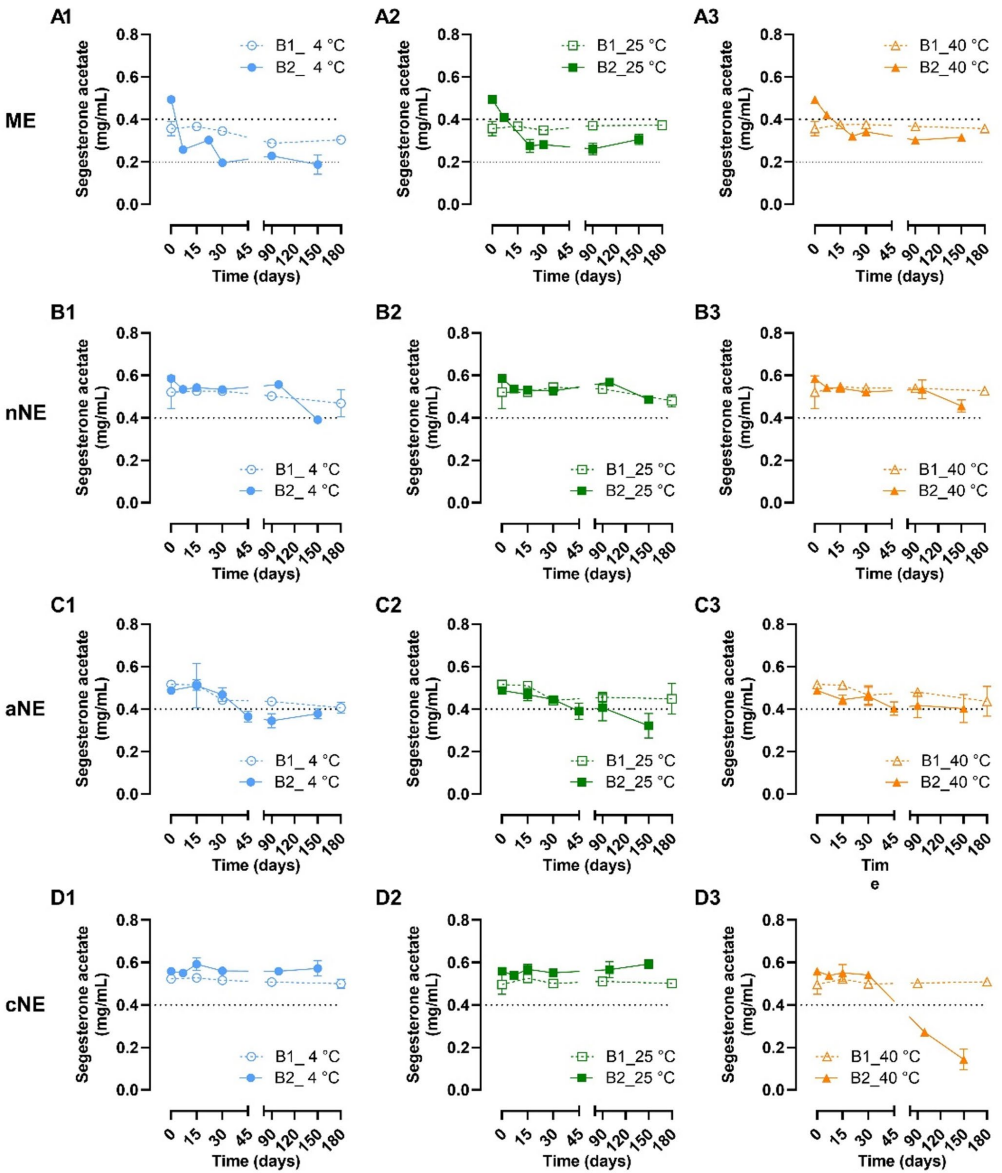
Chemical stability of segesterone acetate in two different batches (B1 and B2) of the selected microemulsion (ME), neutral nanoemulsion (nNE), anionic nanoemulsion (aNE) and cationic nanoemulsion (cNE) evaluated over 180 days after preparation. Segesterone acetate concentrations are presented as mean ± standard deviation (*n = 3*)

The concentration of SA was also evaluated up to 180 days after preparation of the two batches of the ME, nNE, cNE and aNE stored at three different temperatures (Fig. 3), reflecting both chemical stability and physical stability in terms of drug precipitation. Except for the ME stored at 4 °C, in B1, with lower initial SA concentration, there was no significant change of the SA concentration in the ME (Fig. 3A1 to A3); while in the second batch (B2), with a higher initial SA concentration, SA levels decreased over the first 30 days of preparation, especially when stored at lower temperatures.

Regarding the stability of SA in the nanoemulsions (Fig. 3B1 to D3), generally, SA concentrations remained stable until, at least, 90 days after preparation. After that, some fluctuations between the two prepared batches of nNE, aNE, and cNE were also observed. In particular on the batch B2 of the cNE stored at 40 °C, a sharp decrease of SA concentration was observed from 90 days further.

### Antimicrobial activity of the nanoemulsion

Regarding the microbiological preservation of the formulations, from our experience, the concentrated nNE vehicle did not develop visible microbial contamination for several weeks without the addition of antimicrobial preservatives. Nevertheless, to know whether it would comply with future regulatory requirements, that assumption required formal testing. Since cetalkonium chloride could also provide and additional antimicrobial effect to the NE, the cNE vehicle was also tested. However, upon formal testing, neither the diluted nNE nor a cNE fulfilled all the criteria of the pharmacopeial test “5.1.3. Efficacy of antimicrobial preservation” to be considered itself as having adequate antimicrobial activity requested for preservation of nasal preparations (and ear preparations, preparations for cutaneous application and preparations for inhalation) (Table 3). Nevertheless, they did lead to a strong 5 to 6-fold Log_10_ reduction of *S. aureus* and *C. albicans*. In view of these results, it was likely that the aNE and the ME vehicles would also not pass the test, so we did not test them.

**Table 3:** Challenge test results presented as logarithm of colony forming units per gram [Log_10_(CFU/g)] and its reduction over time after inoculation

Day		S. aureus			*P*. aeruginosa		C. albicans		A. brasiliensis	
		nNE	cNE		nNE	cNE	nNE	cNE	nNE	cNE
**0**	**Log**_10_ **(CFU/g)**	6.1	6.1		6.1	6.11	5.24	5.24	4.94	4.94
**2**	Log_10_(CFU/g)	1.2	0.7		6.4	5.28				
	Log_10_ reduction	5	5		0	1				
	Criteria A (Min. Log_10_ reduction)	2	2		2	2				
	Passed criteria?	**Yes**	**Yes**		**No**	**No**				
**7**	**Log**_10_ **(CFU/g)**	0	0		6.5	6.48				
	Log_10_ reduction	6	6		0	0				
	Criteria A (Min. Log_10_ reduction)	3	3		3	3				
	Passed criteria?	**Yes**	**Yes**		**No**	**No**				
**14**	**Log**_10_ **(CFU/g)**	0	0		6.5	6.5	0	0	4.37	3.59
	Log_10_ reduction	6	6		0	0	5	5	1	1
	Criteria A (Min. Log_10_ reduction)						2	2	2	2
	Passed criteria?						**Yes**	**Yes**	**No**	**No**
	Criteria B (Min. Log_10_ reduction)	3	3		3	3	1	1	1	1
	Passed criteria?	**Yes**	**Yes**		**No**	**No**	**Yes**	**Yes**	**Yes**	**Yes**

Since the excipients of the nNE have no antimicrobial effects previously described in literature, the antimicrobial effects of the **nNE** vehicle were then further evaluated by determining the MIC and MLC values (Table 4), which were, respectively, 0.05% and 0.1% (preconcentrate concentration, w/v) in both *S. aureus* and *C. albicans*. For *A. brasiliensis*, although the MIC was similar, the MLC was much higher (12.5%, w/v), suggesting a possible fungistatic effect. For P. aeruginosa, no antimicrobial activity was observed as both MIC and MLC values were above 25% (w/v).

**Table 4:** Antimicrobial effects of the neutral nanoemulsion (nNE) tested in different microorganisms expressed as minimal inhibitory concentration (MIC) and minimal lethal concentration (MLC), corresponding to preconcentrate (PC) final concentration (% w/v)

		S. aureus and C. albicans		*P*. aeruginosa and E. coli		A. brasiliensis	
		N 1	N 2	N 1	N 2	N 1	N 2
**MIC** (PC %, w/v)	Visual analysis	0.05	0.05	> 25	> 25	0.05	0.05
	Resazurin reduction	0.05	0.05	> 25	> 25	n.p.	n.p.
**MLC** (PC %, w/v)		0.1	0.1	> 25	> 25	12.5	12.5

n.p., not performed

### In vitro safety and permeation of selected formulations

**Fig. 4:**
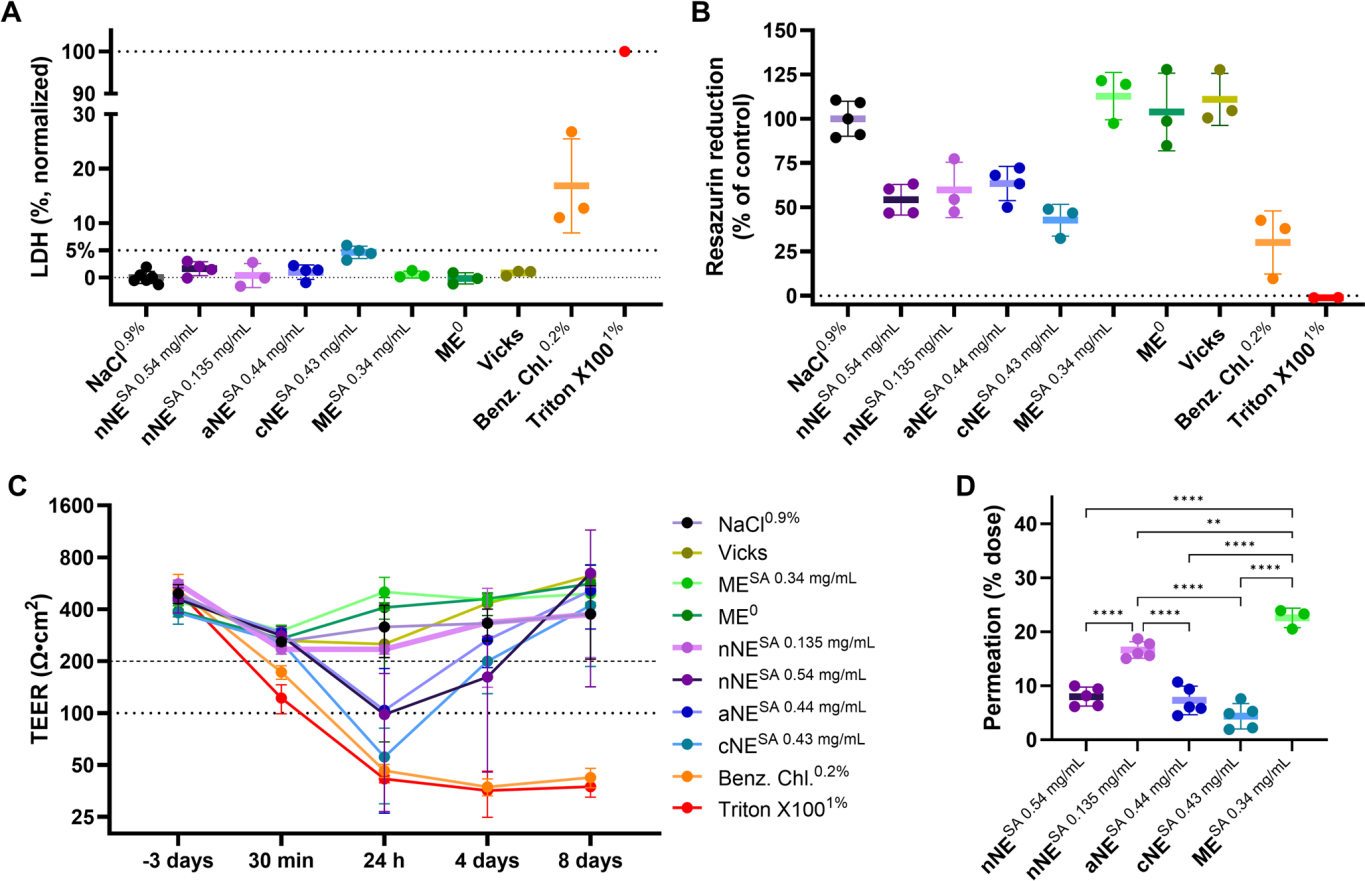
In vitro safety and permeation on MucilAir™ tissue model. The tested formulations were a microemulsion (ME) loaded (0.34 mg/mL) or not with segesterone acetate (SA), a neutral nanoemulsion (nNE) with two SA concentrations (0.54 mg/mL, and the same diluted 4-fold to 0.135 mg/mL), an anionic nanoemulsion (aNE) with SA at 0.44 mg/mL, and a cationic nanoemulsion (cNE) with 0.43 mg/mL of SA. Safety was assessed by lactate dehydrogenase (LDH) release quantification (**A**), resazurin reduction (**B**) and transepithelial electrical resistance (TEER) measurement over time (**C**). The effects of each formulation and SA concentrations on its permeation across this tissue are also studied (**D**). Triton X-100 1% was used as positive toxicity control; benzalkonium chloride 0.2% as highly irritating control; Vicks as a commercial product control; and NaCl 0.9% as a negative toxicity control. The indicated SA levels were obtained by HPLC assay. Data is presented as mean ± standard deviation, and SA permeation results were statistically analyzed by a one-way ANOVA (**p < 0.01; ****p < 0.0001)

The in vitro safety of the selected formulations was first evaluated on the human nasal tissue model MucilAir™, with the results being depicted on Fig. 4. Since LDH release is a measure of cytotoxicity that is directly correlated with the number of lysed cells in culture, the present results show that none of the formulations tested, whether or not containing SA, caused significant damage to the plasma membranes of MucilAir™ cells. In fact, only cNE with 0.43 mg/mL of SA caused a slightly increased LDH release (around 5% of the total release caused by Triton X-100 1%, Fig. 4A). However, when the cells were in contact with the nNE, cNE and aNE containing different concentrations of SA, a reduction in their metabolic capacity (resazurin reduction) to around half of normal was observed, particularly with cNE, something that did not occur after the cells were incubated with ME with and without SA (Fig. 4B). That might be in accordance with the decrease of MucilAir™ tissue TEER obtained at different times after formulations incubation. In fact, tissue integrity (or the permeability barrier) decreased 24 h after being in contact with the nanoemulsions at higher concentrations (TEER < 200 Ω·cm^2^), with a substantial decrease occurring after incubation with the cNE (TEER < 100 Ω·cm^2^). Still, that decrease was not so evident when a less concentrated nNE [with a lower preconcentrate and SA concentration (0.135 mg/mL)] was tested, indicating a dose-dependent effect. Therefore, if the neuroprotective effects can be attained using a low SA dose, this tested nNE might indeed be a safe formulation. Nevertheless, 8 days after MucilAir™ contact with the different nanoemulsions, TEER values normalized to above 200 Ω·cm^2^, indicating a somehow reversible influence in tissue integrity or permeability, possibly correlated with the metabolic impact that these formulations had on these cells. As for ME, it has not caused any decrease of tissue integrity, which is consistent with both LDH release and resazurin reduction results.

The influence of the different formulations studied on SA permeation through the MucilAir™ model was also studied (Fig. 4D). The SA permeation in terms of percentage of the total incubated dose attained 30 min after ME incubation was significantly higher than the permeation obtained with the cNE, aNE, nNE (*p* < 0.0001), and the nNE containing a lower SA concentration (*p* < 0.01). The cNE resulted in the lowest SA permeation. The permeation of SA from aNE and from nNE with the higher SA concentration was also lower than 10% of the total incubated dose. Still, when the initial concentration of SA in the nNE was lower (0.135 mg/mL), SA relative permeation was significantly higher than with the other nanoemulsions containing higher SA concentrations (*p* < 0.0001), suggesting a saturation effect with higher SA doses.

**Fig. 5:**
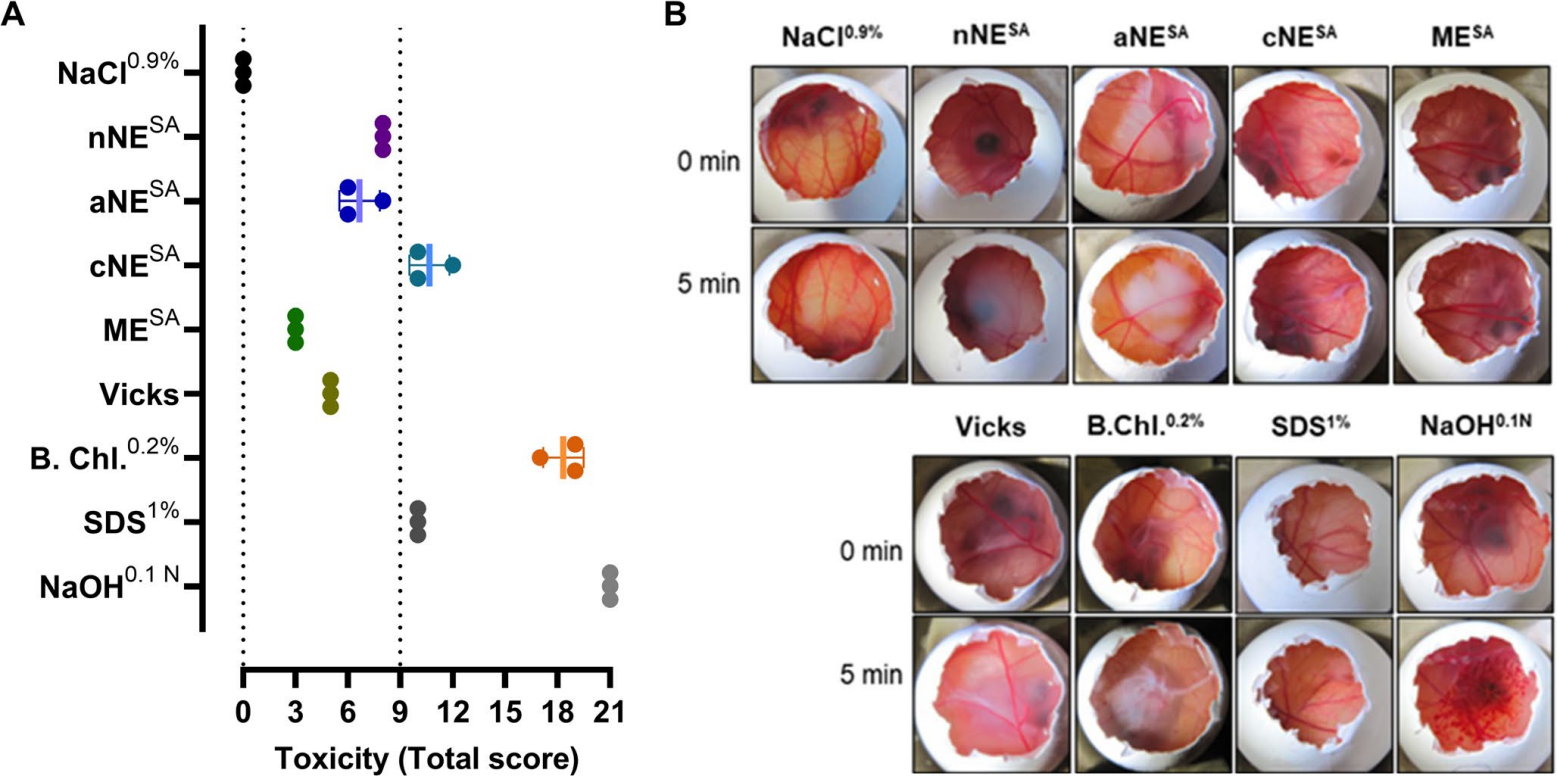
HET-CAM in vitro results showing the irritancy potential of each tested formulation - neutral nanoemulsion (nNE), anionic nanoemulsion (aNE), cationic nanoemulsion (cNE) and microemulsion (ME) – containing segesterone acetate (SA), the negative [sodium chloride (NaCl) 0.9%], reference substances (Vicks nasal medicine and benzalkonium chloride 0.2%) and positive controls [sodium dodecyl sulfate (SDS) 1% (w/v) and sodium hydroxide (NaOH) 1 N]. (**A**) Mean ± standard deviation of the obtained scores 5 min after formulations contact with chorioallantoic membrane of chicken eggs (*n = 3*). (**B**) Representative HET-CAM image records of one egg per tested group right before each test substance addition, and at the end of the test (5 min)

The in vitro irritancy potential of each selected formulation (nNE, aNE, cNE and ME, the exact same batches that were used on MucilAir™), was also assessed by the HET-CAM assay (Fig. 5). Control substances (SDS 1% and NaOH 0.1 N as positive controls, NaCl 0.9% as negative control) and reference substances (Vicks nasal medicine and benzalkonium chloride 0.2%) were used for comparison purposes. ME was classified as non-severely irritating, showing the lowest irritancy score of all the tested formulations (3, Fig. 5A), supporting the results obtained on the MucilAir™ model. In fact, ME only led to a very slight lysis 2 min after contact with the eggs’ chorioallantoic membranes (Fig. 5B). As for the nanoemulsions, both the nNE and the aNE were also classified as non-severely irritating, with the obtained scores being 8 and 7 ± 1, respectively (Fig. 5A). In fact, the nNE caused vascular lysis and hemorrhage to be observed 2 min after being in contact with chorioallantoic membranes, whereas the aNE caused vascular lysis after 2 min, and hemorrhage after 5 min (Fig. 5B). On the other hand, the cNE was classified as severely irritating with an obtained score of 11 ± 1 (Fig. 5A), causing vascular lysis and hemorrhage only after 30 s in contact with the membranes (Fig. 5B). However, as also observed in Fig. 5, the irritancy score of cNE was substantially lower than the score obtained after contact with benzalkonium chloride 0.2% (score of 18 ± 1), a well know irritant solution, here used at a 10-fold higher concentration than usually used to preserve nasal preparations.

**Fig. 6:**
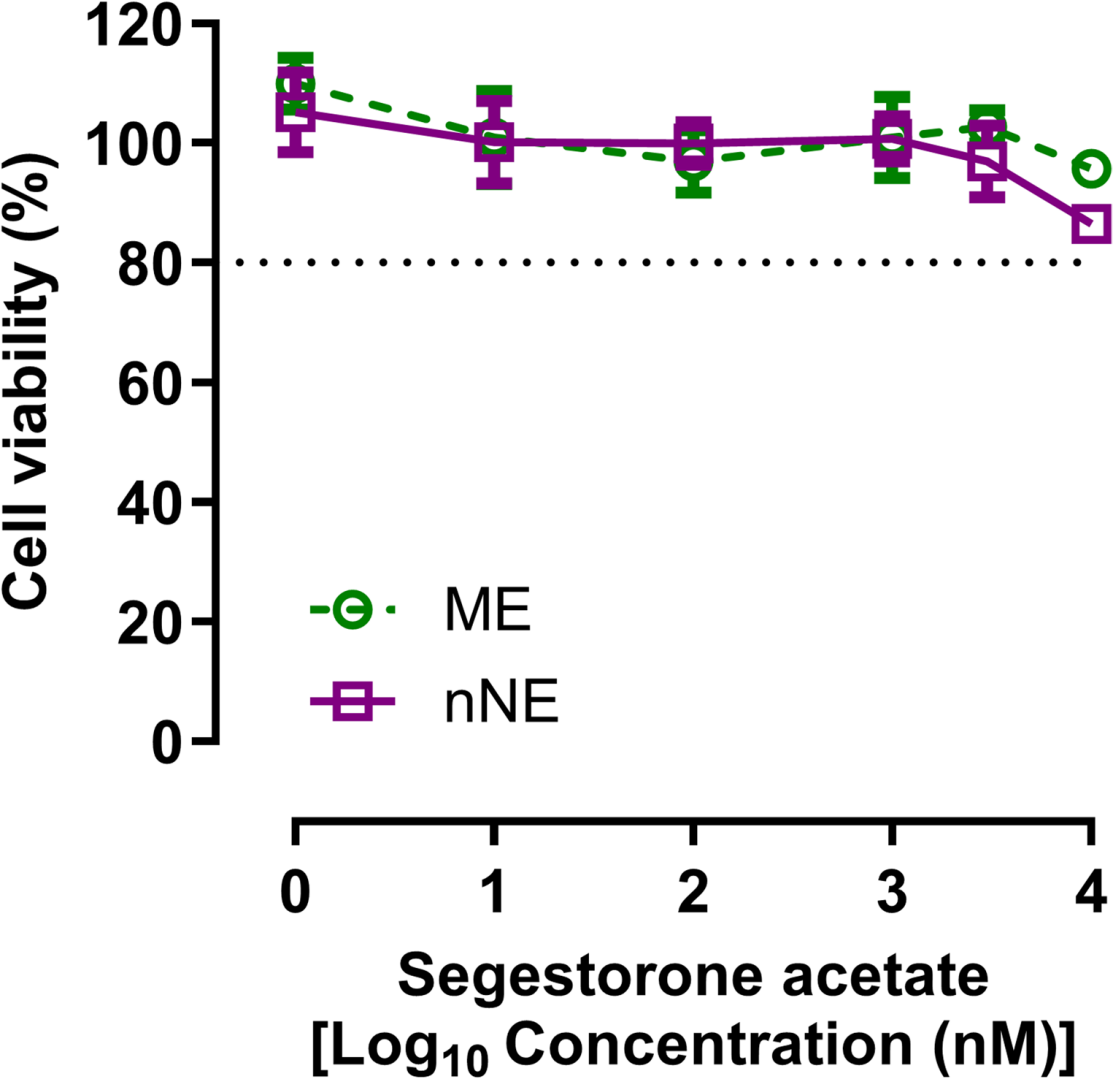
In vitro cytotoxicity in primary cultures of cortical cells of the nNE and ME containing segesterone acetate at different concentrations (0–10000 nM) evaluated after 24 h of contact with the formulations. The time axis is presented in the form of a log base 10 scale, and cell viability is expressed as percentage of the viability of control. The results are expressed as percentage relative to the absorbance of control and presented as mean ± standard deviation *(n = 3)*

Considering the safety results obtained on MucilAir™ and HET-CAM tests, the acute cytotoxicity potential in brain tissue of the ME and one representative NE (the nNE) was further evaluated in mixed primary cultures of neurons and glial cells. This in vitro evaluation was performed assuming that, in the worst-case scenario, the formulations are absorbed by the cells in the same proportion as the drug. The safety of the nNE and ME was demonstrated up to at least 1000 nM (3705 ng/mL) of SA, which are concentrations values much higher than those expected to be attained in the brain (Fig. 6).

In addition, the safety of the nNE for human skin was confirmed in a reconstituted human epidermis model at the target SA concentrations of 0.48 mg/g and 11.2 mg/g (Supplementary data, Section S4).

### In vivo safety of selected formulations

**Fig. 7:**
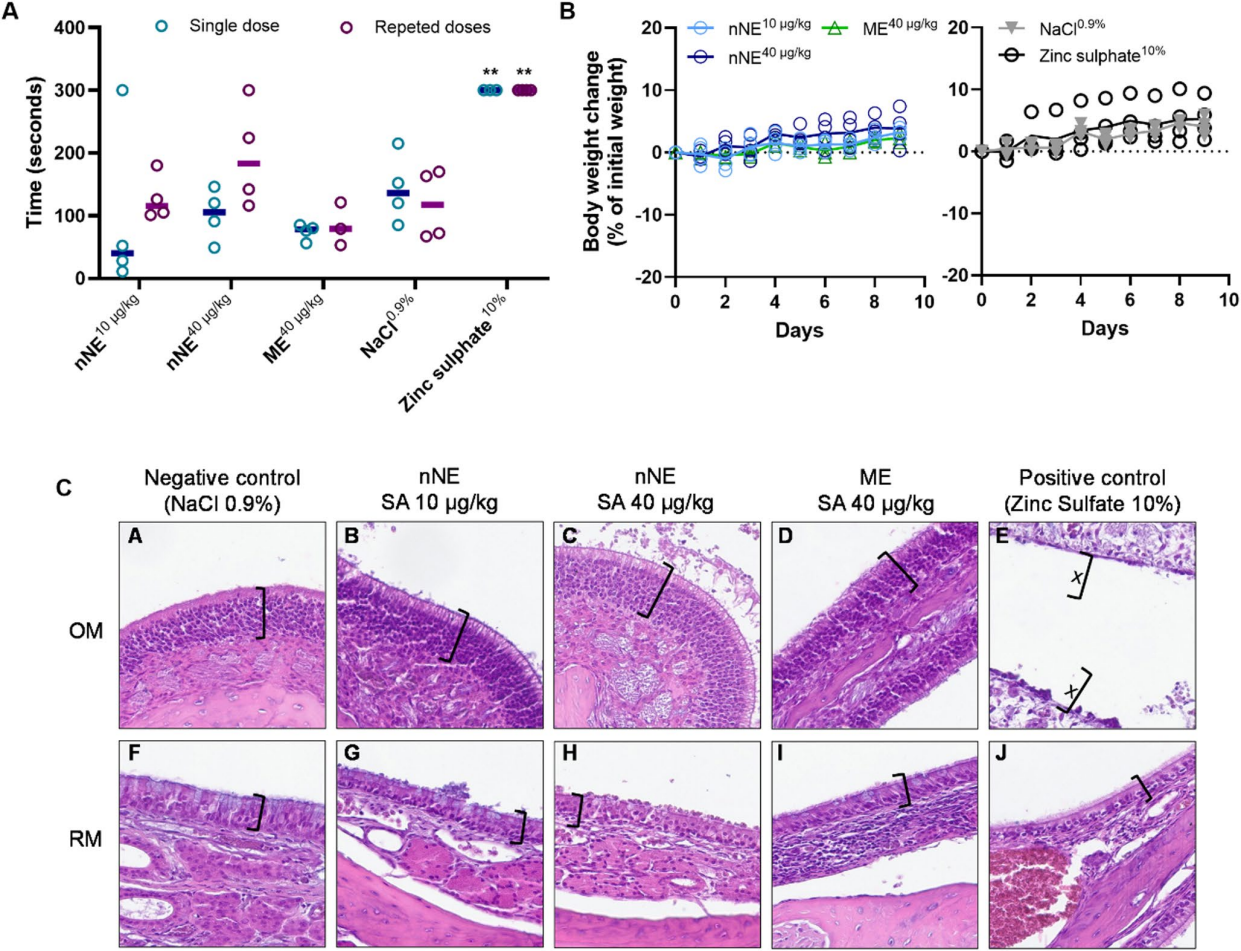
In vivo safety results of the microemulsion (ME) and neutral nanoemulsion (nNE) after a single and a repeated intranasal administration scheduling. Two segesterone acetate (SA) doses were tested using the nNE (10 and 40 µg/kg) and one SA dose was administered using the ME (40 µg/kg). (**A**) Buried food seeking test results. Statistically significant differences between the results obtained in the group that received intranasal NaCl 0.9% and other test groups were evaluated by a two-way ANOVA with Dunnett’s post hoc test (**p < 0.01). (**B**) Body weight changes over repeated daily doses. (**C**) Images of histological structures of nasal mucosa sections after 7 days of treatment (x200 magnification). OM, Olfactory mucosa; RM respiratory mucosa; ] Epithelial layer; x] Epithelial layer strongly denuded

The in vivo safety of the ME (SA dose of 40 µg/kg) and the nNE (two different SA doses, 10 and 40 µg/kg) was evaluated in rats after their intranasal administration in a single and repeated dose regimen. The safety was evaluated by assessing the loss of olfactory function in rats using the buried food seeking test and by evaluating weight changes throughout the repeated dose schedule. Both single and multiple intranasal doses of SA at 40 µg/kg using the ME did not increase the time that starved rats took to find the pellet food (Fig. 7A). When a single SA dose of 10 µg/kg or 40 µg/kg was administered using the nNE, the time the rats took to find the pellet was also not different, even being apparently slightly shorter than those treated with NaCl 0.9%. Only a slight non-significant increase in that time occurred after 7 consecutive days of nNE administration at the highest SA dose, indicating that the nNE did not compromise olfactory function of rats after multiple administrations of a higher SA dose.

The weight of the animals remained stable throughout the 7 days of dosing in the three groups tested, with a normal gradual increase being observed and explained by the natural growth of the animals. Therefore, neither the formulations nor the different SA doses proved to have an impact on the normal growth process of the rats (Fig. 7B).

In agreement with the olfaction study results, the histological analysis of the rats’ nasal cavities previously subjected to the repeated dose scheduling revealed no changes in the olfactory nor in the respiratory mucosa. In fact, Fig. 7C clearly demonstrates that, contrary to the complete destruction of the olfactory mucosa caused by the intranasal administration of zinc sulfate 10% (a solution well known for damaging this epithelium type), the integrity of the olfactory mucosa was maintained after the 7 days of ME or nNE administration. Likewise, the formulations tested did not cause any visible changes in the respiratory mucosa where the epithelial layer thickness was similar to the one observed in rats treated with 0.9% NaCl.

### Brain and plasma segesterone acetate levels after intranasal administration of selected formulations

To understand the impact that each of the selected formulations had on the effective SA delivery from the nasal cavity to brain tissue and/or systemic circulation, SA concentrations in the plasma and brain tissue of rats that received an intranasal dose of each formulation were assessed 30 and 60 min after administration.

**Fig. 8:**
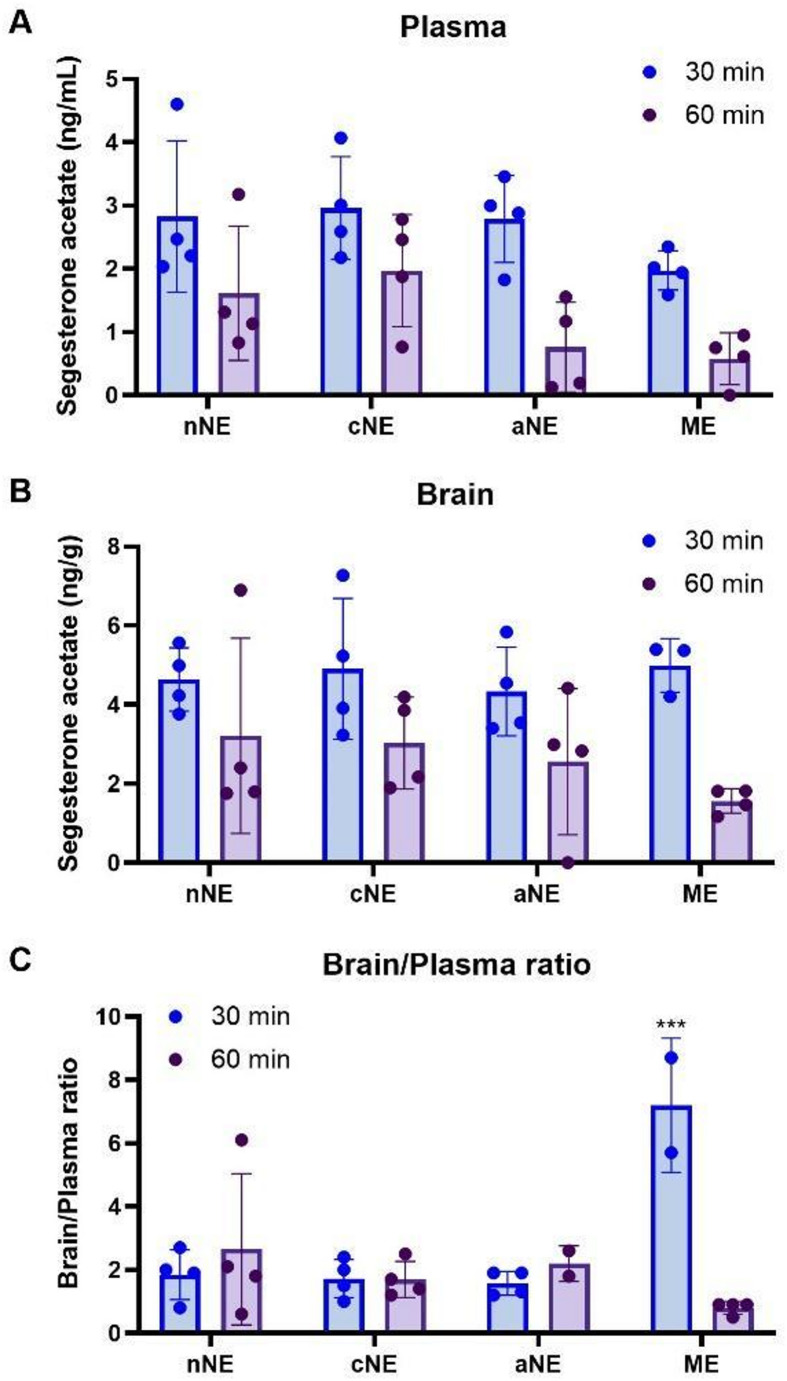
Plasmatic (**A**) and brain tissue (**B**) concentrations of segesterone acetate 30 and 60 min after intranasal administration to rats (*n = 4*) of each formulation strategy that fulfilled the quality target product profile – neutral nanoemulsion (nNE), anionic nanoemulsion (aNE), cationic nanoemulsion (cNE) and a microemulsion (ME). Brain/plasma ratio (**C**) was also calculated, and values statistically analyzed by a two-way ANOVA with Sidak’s post-hoc test (***p < 0.001)

Generally, there were no significant differences in plasmatic and cerebral SA concentrations after the administration of all formulations using a SA dose of 40 µg/kg (Fig. 8). Particularly after 30 min of administration, only SA plasma concentrations were slightly lower after ME administration compared with those obtained with all tested nanoemulsions, justifying the statistically higher brain/plasma ratio (*p* < 0.001) obtained with this strategy.

## Discussion

The NE compositions compared in the present work are variants of a NE that share an uncommon composition with a high proportion of hydrophobic components to hydrophilic surfactant (5:1 mass ratio) [27]. Among the different NE strategies compared in this work, the nNE was the most homogeneous one (PDI < 0.1). It should be emphasized that, despite the high lipid content, such homogeneity is obtained using a low energy preparation method (phase inversion composition), which is not usually seen [36]. That method is important to obtain a high drug loading of liposoluble drugs (here ~ 22 mg/g of SA in the preconcentrate) and, therefore, facilitate formulation translation and, later on, scale transposition.

The increase in the nNE droplet size over time at room temperature and at 40 °C was likely due to the fact that PEG, used to prepare the initial NE (with 50% aqueous phase, 11.2 mg/g SA), was not present in the saline used to further dilute the NE to the final SA concentration (~ 0.48 mg/g). In fact, it has been previously demonstrated that the mean size and PDI of the nNE prepared in water (without polymers such as PEG in the aqueous phase) reversibly changed droplet size upon refrigeration compared to room temperature, demonstrating the temperature sensibility that is characteristic of this particular composition [27]. Therefore, this physical instability of the diluted nNE might have been prevented if PEG would have also been added to the saline solution used to dilute the nNE to the final desired SA concentration. Nevertheless, PDI remained consistently very low over time at both 4 °C and room temperature, and only in the accelerated condition of 40 °C there was a clear progressive destabilization of the nNE. The cNE was stable over time at all three temperatures, likely due to the electric repulsion effect (zeta potential above 20 mV). However, the aNE was only stable at 4 °C, despite the zeta potential below − 20 mV, meaning that the charged polymer, in the present conditions, was likely not conveniently adsorbed at the NE droplets interface to prevent their coalescence. With respect to the ME, it was not clear whether the increase in measured mean size and PDI, even under refrigeration, was influenced by excess drug precipitation in the very diluted formulation. Nevertheless, size remained under 100 nm, and PDI below 0.3, which cannot be considered as very problematic (once the precipitation issue is resolved). The high potency of SA makes it a very suitable candidate for intranasal administration. This feature also dictated that the nNE and the ME developed in the present work required a high proportion of aqueous phase, which resulted in them having low irritancy profiles, even in the HET-CAM model that is a surrogate of ophthalmic irritation potential. Even though this test is not commonly used to evaluate the safety of nasal preparations, it has been used at least by Muke at al. with that purpose [37]. In addition, it has also been used as a surrogate for other mucosae, such as the vaginal tissue [38]. Indeed, ophthalmic preparations drain to the nasal cavity, and no concerns are usually raised regarding possible toxicity of ophthalmic preparations for the nasal mucosa. Interestingly, the only formulation scoring as severely irritating in that test, the cNE, was the only inducing a transient TEER reduction to values under 100 Ω·cm^2^, a slight increase of relative LDH release of about 5%, and a decrease in resazurin reduction over 50%. On the other hand, the formulations with the lowest irritancy scores in the HET-CAM model (the ME and the reference formulation Vicks) had no measurable effects on the in vitro nasal respiratory mucosa model. This means that it might be worth investing in validating the simple HET-CAM model as an in vitro screening test of the safety of intranasal formulations, even though other tests with more specific outputs might be complementary used, such as the effects of formulations on nasal ciliary function [39, 40], which we have not addressed in our work.

Considering the histological evaluation and olfactory function tests, the ME and nNE formulations were also safe after repeated intranasal administration to rats. In particular, the present ME composition has indeed very safe excipients (safer than the NE) and could therefore be used in a less diluted form, in the future, or even administered as a SMEDDS as demonstrated before [29].

Importantly, the future reduction of the initial concentration of SA in the ME preconcentrate would likely solve the drug precipitation issue found at long term storage with this formulation. Since it is known that SA is a highly stable molecule [41], and considering that the initial concentration of SA in the SMEDDS (ME preconcentrate) was close to saturation, the decrease of SA concentration with storage time is likely to be explained by the precipitation of the excess of SA in the ME, particularly in B1 at room temperature and under refrigeration. Thus, increasing the excipients-to-drug proportion in the ME, or even its replacement by the SMEDDS form, is expected to better solubilize SA and prevent its precipitation. Furthermore, the use of the SMEDDS form would make sterilization and unidose unnecessary because it would not be prone to microbiological growth. In fact, similar SMEDDS vehicles were already successfully tested for the intranasal delivery of perampanel (an antiepileptic molecule) and simvastatin [28–30]. Even though the brain SA levels obtained with the SA-ME were not distinguishable from those obtained with the different NE approaches, the intranasal administration of the SA-ME resulted in higher brain/plasma ratios at 30 min after administration compared with the intranasal administration of any of the NE. This could possibly be explained by the small size of the ME droplets, making them able to promote a more efficient transport of SA molecules (free or encapsulated in the nanometric droplets) by direct routes to the brain.

Considering SA delivery, the SA plasma and brain levels here reported (approximately 3 and 5 ng/g, respectively, 30 min post-administration) cannot be directly compared to the results previously obtained with a proprietary oleogel formulation of high viscosity (approximately 4 and 13 ng/g, respectively, 1 h post-administration) [6]. In fact, in the present study, half the dose was administered, rats were used instead of mice, and the early time point of 30 min was not assessed in the previous work using the oleogel. Nevertheless, given the lower viscosity of our formulations, which possibly leads to an increased diffusion of SA, a faster absorption can be expected and, overall, the SA levels seem to be in good agreement with those obtained before, taking in consideration the lower dose. As in the previous study by Fréchou et al. [6] there was a reduction in infarct volume by 32% (*P* < 0.01), we can easily expect that therapeutic levels can be obtained with the present formulations as well, particularly considering that is very easy to adjust the final drug strength if necessary by preparing less diluted formulations. Additionally, and against our initial expectations, neither the more viscous aNE, nor the positively charged cNE led to a greater passage of SA to the brain or bloodstream. We hypothesize that the absorption is so fast that the increased retention that could be provided by the cationic or anionic NE make no significant difference. Since the cNE was apparently more toxic and the aNE less stable than the nNE, among the NE strategies, the nNE could be considered the most promising strategy to proceed to future work.

For future development of an SA-nNE or SA-ME spray for intranasal administration, since the microbiological preservation cannot be guaranteed (unless ME was used as a SMEDDS), sterilization might be required. Interestingly though, the nNE vehicle has strong bactericidal and fungicide effects (at least in some species, like *S. aureus* and *C. albicans*), which could be explored in the future for different applications. This antimicrobial effect was attributed mainly to Capryol 90 (propylene glycol monocaprylate, data not shown), which is chemically related with substances like caprylic acid or glycerol monocaprylate that have been previously demonstrated by other authors to have antimicrobial effect [42–45].

## Conclusion

Taking into consideration all the data, the nNE composition reported in this work is a simple composition with adequate pharmaceutical and safety attributes, which supports its further development as a formulation strategy for SA intranasal delivery. In addition, and as an alternative, the ME composition could be optimized to overcome its physical stability issues, observed in long-term storage. In particular, the increased brain/plasma ratio obtained with the ME strategy at 30 min post-administration deserves further consideration in future studies.

## Electronic supplementary material

Below is the link to the electronic supplementary material.

Supplementary Material 1

## Data Availability

No substantial data sets are associated with the present work. Raw data or other omitted experimental details are available upon request.
