# Supplementary material for: Optimized intranasal delivery of segesterone acetate progestin to the brain using nanoemulsions and microemulsions
Source: Drug Deliv Transl Res. 2025 Jun 18;15(12):4795–818. doi: 10.1007/s13346-025-01897-7 (PMC12619833; doi:10.1007/s13346-025-01897-7)
Supplement: Supplementary file 1 — Supplementary Material 1 [file 13346_2025_1897_MOESM1_ESM.pdf]

Optimized Intranasal Delivery of Segesterone Acetate Progestin to the Brain Using Nanoemulsions and Microemulsions

Carina Peres[1], Sara Meirinho[1], Samille Ferreira da Silva[1], Izamara Maocha[1], Anabela Chiangalala[1], Susana Alves Ferreira[1], Márcio Rodrigues[1,2], Shimin Zhang[3], Narender Kumar[3], Regine Sitruk-Ware[3], Rui Caetano Oliveira[4,5], Carlos Gaspar[6], Ana Palmeira-de-Oliveira[6], Rita Palmeira-de-Oliveira[6], Gilberto Alves[1,2], Graça Baltazar[1], Adriana O. Santos[1,*]

[1] RISE-Health, Faculty of Health Sciences, University of Beira Interior, 6200-506 Covilhã, Portugal

[2] BRIDGES - Biotechnology Research, Innovation and Design for Health Products, Polytechnic University of Guarda, 6300-559 Guarda, Portugal

[3] Population Council, Center for Biomedical Research, 1230 York Avenue, New York, NY, 10065, USA

[4] Pathology Department, Centro Hospitalar e Universitário de Coimbra, Coimbra, Portugal; Biophysics Institute, Faculty of Medicine, University of Coimbra, 3000-548 Coimbra, Portugal;

[5] Institute for Clinical and Biomedical Research (iCBR) Area of Environment Genetics and Oncobiology (CIMAGO), Faculty of Medicine, University of Coimbra, 3000-548 Coimbra, Portugal.

[6] Labfit-HPRD, Health Products Research and Development Lda, 6200-284 Covilhã, Portugal.

Supplementary information

S1. Initial formulation composition screening

The initial neutral NE containing phosphate-buffered saline as aqueous phase (nNE^{PB}) had a PDI > 0.2 at room temperature. However, at 4 °C, nNE^{PB} demonstrated a PDI < 0.1 and a zeta potential very slightly negative, probably due to the phosphate ions of the saline buffer used as aqueous phase (**Table S1**). Since the phosphate buffer with 0.6% NaCl is hypotonic, and once NE preconcentrate did not significantly increase the final osmolality (data not shown), the NaCl concentration used in the aqueous phase was increased to 0.72% to achieve isotonicity. Still, as also shown in **Table S1**, osmolality values did not significantly change, contrary to size and PDI that revealed to be lower and zeta potential being slightly closer to neutrality.

Table S1 Characterization of neutral and cationic nanoemulsions of segesterone acetate (SA) prepared in buffered saline aqueous phases at room temperature (RT) and after refrigeration (4 °C).

Code name	Target [PC] (%)	Target [SA] (mg/g)	T (°C)	Aqueous phase	Z-Ave (nm)	PDI	Zeta potential (mV)	Aqueous phase osmolality (mOsmol/Kg)
**nNE^{PB}**	2.6	0.58	RT	PB+NaCl 0.6%	130	0.249	n.d.	
	2.6	0.58	**4 °C**	PB+NaCl 0.6%	107	0.086	-12.4	239
	2.6	0.58	4 °C	PB+NaCl **0.72%**	78.5	0.065	n.d.	
	2.1	0.48	4 °C	PB+NaCl 0.72%	74.4	0.026	-7.84	280
**cNE^{PB}**	2.6	0.58	**RT**	PB+NaCl 0.6%	98.4	0.051	n.d.	
	2.6	0.58	**4 °C**	PB+NaCl 0.6%	97,1	0.079	n.d.	239
	2.6	0.58	**RT**	PB+NaCl **0.72%**	87.7	0.086	17.4	
**$2cNE^{PB}$**	2.1	0.48	4 °C	PB+NaCl 0.72%	304	0.258	17.7	280

n.d., not determined, PC, preconcentrate; PB, Phosphate buffer 20 mM; PDI, polydispersity index; SA, Segesterone acetate; T, temperature before dilution for measurement; Z-Ave, mean hydrodynamic size

Contrary to the nNE^{PB}, its cationic version (cNE^{PB}) had mean sizes < 100 nm and PDI < 0.1 at both room temperature and 4 °C. The zeta potential was positive, but not as positive as it could be expected, probably due to the presence of NaCl and phosphate ions (Table S1). This fact led to the decision to remove the phosphate buffer from the aqueous phase and trying to use a higher proportion of cationic lipid (up to 2% in the preconcentrate, $2cNE^{PB}$). However, this strategy revealed a high increase in the size and PDI of NE, being no further pursued.

That leads us to conclude that the amount of cationic lipid in the NE preconcentrate is critical and should be kept at a maximum of 1% in the preconcentrate composition.

A SA-ME was also prepared using phosphate buffer and NaCl 0.72%. It showed a droplet size of about 19 nm and PDI < 0.1 (**Table S2**), being those attributes consistent with the previously obtained [1]. As for the osmolality, it was a bit increased mostly due to the presence of a large percentage of Transcutol®, which is a permeable solute and therefore does not contribute to tonicity of the preparations. Still, the osmolality of the prepared ME was within a safe range.

Table S2 Characterization of a neutral microemulsion (ME) of segesterone acetate (SA) prepared in buffered saline aqueous phase. Z-Ave and PDI data are mean ± standard deviation of 2 independent batches.

**Code name**	**Target [PC] (%)**	**Target [SA] (mg/g)**	**T (°C)**	**Aqueous phase**	**Z-Ave (nm)**	**PDI**	**N**	**Osmolality (mOsmol/Kg)**
**ME**	2.6	0.48	RT	PB+NaCl 0.72%	19.3 ± 0.4	0.072 ± 0.012	2	432 ± 28

n.d., not determined, PC, preconcentrate; PDI, polydispersity index; PB, Phosphate buffer 20 mM, RT, Room temperature; T, temperature before dilution for measurement; Z-Ave, mean hydrodynamic size

Considering the obtained size heterogeneity of the nNE at room temperature, a PEG 4000 solution (with 1 or 4% PEG) was used as aqueous phase in concentrated NE (with 50% aqueous phase) to make it more homogeneous [2]. After different dilutions in saline (NaCl 0.9%), the mean droplet size was mostly around 80 to 100 nm, the PDI < 0.1 (between 0.04 and 0.08), and zeta potential was neutral (**Table S3**). Since glycerol 2.6% can also be used as an isotonic agent, the dilution of nNE with glycerol 2.6% was tested, with mean droplet sizes and PDI values being maintained similar to the ones obtained using NaCl 0.9%. Neither the presence of segesterone acetate (SA), the extent of dilution, or filter sterilization had a relevant effect on the attributes of the nNE formulations (**Table S3)**.

Table S3 Characterization of neutral nanoemulsions prepared with unbuffered aqueous phase, at room temperature (the day of preparation).

**Code name**	**Concentrated NE: 50% PC**		**Dilution of the concentrated NE**			**Without filtration**				**After filter-sterilization**			
	**Z-Ave (nm)**	**PDI**	**Target [SA] (mg/g)**	**Target [PC] (%)**	**Aq. phase**	**Z-Ave (nm)**	**PDI**	**Zeta potential (mV)**	**SA assay (mg/mL)**	**Z-Ave (nm)**	**PDI**	**Zeta potential (mV)**	**SA assay (mg/mL)**
**nNE$^{PEG\ 1\%}$**	n.d.	n.d.	0.48	2.1	NaCl 0.9%	98.0	0.079	w.q.	n.d.				
**nNE$^{PEG\ 4\%}$**	n.d.	n.d.	**0.25**	**1.1**	Glycerol 2.6%	97.4	0.066	w.q.	n.d.				
	n.d.	n.d.	**0.48**	**2.1**	Glycerol 2.6%	99.1	0.053	w.q.	n.d.				
	n.d.	n.d.	**0.80**	**3.6**	Glycerol 2.6%	102	0.049	w.q.	n.d.				
	n.d.	n.d.	**0**	**2.1**	NaCl 0.9%	81.0	0.057	-7.37	n.d.				
	n.d.	n.d.	**0.25**	**1.1**	NaCl 0.9%	89.5	0.071	-7.21	n.d.				
	n.d.	n.d.	**0.80**	**3.6**	NaCl 0.9%	97.6	0.041	-2.46	n.d.				
	n.d.	n.d.	**0.48**	**2.1**	NaCl 0.9%	90.4	0.035	-5.73	n.d.				
	n.d.	n.d.	**0.48**	**2.1**	NaCl 0.9%	103	0.036	-3.80	0.541				
	n.d.	n.d.	**0.48**	**2.1**	NaCl 0.9%	101	0.049	n.d.	n.d.				
	n.d.	n.d.	**0.48**	**2.1**	NaCl 0.9%	87.1	0.032	n.d.	n.d.				
	n.d.	n.d.	**0.48**	**2.1**	NaCl 0.9%	84.8	0.060	n.d.	n.d.				
	n.d.	n.d.	**0.48**	**2.1**	NaCl 0.9%	83.5	0.046	n.d.	n.d.				
	109	0.076	**0**	-	-	-	-	-	-				
	92.8	0.042	0.48	2.1	NaCl 0.9%	93.8	0.034	-8.99	n.d.				
	100	0.051	0.48	2.1	NaCl 0.9%	94.4	0.047	n.d.	0.540				
	102	0.061	0.48	2.1	NaCl 0.9%	83.6	0.047	n.d.	n.d.				
	n.d.	n.d.	0.48	2.1	NaCl 0.9%	120	0.086	-2.20	n.d.	118	0.082	-2.08	0.521
	80.9	0.047	0.48	2.1	NaCl 0.9%	93.8	0.064	n.d.	n.d.	93.2	0.060	n.d.	0.585
	n.d.	n.d.	0.48	2.1	NaCl 0.9%	n.d.	n.d.	n.d.	n.d.	89.2	0.028	n.d.	n.d.
	n.d.	n.d.	0.48	2.1	NaCl 0.9%	n.d.	n.d.	n.d.	n.d.	96.8	0.084	n.d.	n.d.

n.d., not determined, PC, preconcentrate; PDI, polydispersity index; SA, segesterone acetate; w.q., without quality; Z-Ave, mean hydrodynamic size.

The cationic NE (cNE) did not require PEG in the aqueous phase to be small and homogenous (**Table S4**), as we had already observed before [3]. We did get less reproducibility in PDI values between batches thatn with the

nNE though (between 0.047 and 0.276), meaning there must be other uncontrolled factors influencing this attribute. When glycerol 2.6% was used to dilute the cNE, zeta potential became far more positive than the obtained after cNE dilution with NaCl 0.9% (**Table S4**), and therefore it become the preferred aqueous phase for this formulation.

Table S4 Characterization of cationic and anionic nanoemulsions without or with segesterone acetate (SA).

Code name	Concentrated NE: 50% PC		Dilution of the concentrated NE			Without filtration				After filter-sterilization			
	**Z-Ave (nm)**	**PDI**	**Target [SA] (mg/g)**	**Target [PC] (%)**	**Aq. phase**	**Z-Ave (nm)**	**PDI**	**Zeta potential (mV)**	**SA assay (mg/mL)**	**Z-Ave (nm)**	**PDI**	**Zeta potential (mV)**	**SA assay (mg/mL)**
**cNE^{water}**	n.d.	n.d.	0	2.1	NaCl 0.9%	85.0	0.111	6.56					
**cNE^{water}**	n.d.	n.d.	0.48	2.1	NaCl 0.9%	86.2	0.047	11.9	0.551				
**cNE$^{PEG\ 4\%}$**	n.d.	n.d.	0.48	2.1	Glycerol 2.6%	107	0.101	w.q.	0.476				
**cNE^{water}**	n.d.	n.d.	0.48	2.1	Glycerol 2.6%	86.4	0.053	n.d.	n.d.				
**cNE^{Gly}**	98.4	0.231	0.48	2.1	Glycerol 2.6%	83.0	0.121	w.q.	0.495				
	77.7	0.104	0.48	2.1	Glycerol 2.6%	93.3	0.146	n.d.	0.43				
	91.5	0.076	0.48	2.1	Glycerol 2.6%	113	0.276	n.d.	n.d.				
	n.d.	n.d.	0.48	2.1	Glycerol 2.6%	101	0.143	46.2	0.476				
	n.d.	n.d.	0.48	2.1	Glycerol 2.6%	83.9	0.049	n.d.	n.d.				
	n.d.	n.d.	0.48	2.1	Glycerol 2.6%	88.0	0.079	20	n.d.				
	n.d.	n.d.	0.48	2.1	Glycerol 2.6%	90.2	0.135	n.d.	n.d.				
	n.d.	n.d.	0.48	2.1	Glycerol 2.6%	93.3	0.132	33.9	n.d.	91.6	0.133	33.9	0.523
	81.3	0.071	0.48	2.1	Glycerol 2.6%	99.9	0.150	n.d.	n.d.	99.6	0.135	35.5	0.559

n.d., not determined, PC, preconcentrate; PDI, polydispersity index; T, temperature before dilution for measurement; w.q., without quality; Z-Ave, mean hydrodynamic

Aiming to obtain a more viscous and potentially mucoadhesive formulation, aqueous dispersions of Carbopol - an anionic viscosifying agent - were tested to dilute the nNE (with or without PEG), becoming, by this way, an anionic NE (aNE). After adjusting the acidic pH of Carbopol dispersion (pH 4) to a neutral pH value (pH 7), the attributes of the formulations with Carbopol and NaCl (used as isotonizing agent) were not favorable, only obtaining a slight increase in viscosity. For that reason, glycerol 2.6% was instead used as an isotonizing agent (**Table S5**). Carbopol at 0.05% and 0.1% led to a non-significant increase in mean droplet size, but a small increase in PDI was observed, with zeta potential becoming clearly negative (**Table S5**), As it could be suspected, Carbopol was not compatible with the cNE, and led to increased droplet size and PDI values (c+aNE, **Table S5**).

Table S5 Characterization of anionic nanoemulsions. Target SA concentration 0.48 mg/g (PC at 2.1%).

		Without filtration				After filter-sterilization			
**Code name**	**Aq. phase used to dilute the nNE at 50% PC**	**Z-Ave (nm)**	**PDI**	**Zeta potential (mV)**	**SA assay (mg/mL)**	**Z-Ave (nm)**	**PDI**	**Zeta potential (mV)**	**Target SA (mg/g)**
**aNE^{water}**	Glycerol 2.6% + carbopol 0.05%	93.6	0.097	n.d	n.d.				
**aNE$^{PEG\ 4\%}$**	Glycerol 2.6% + carbopol 0.05%	105	0.111	w.q.	n.d.				
	Glycerol 2.6% + carbopol 0.05%	107	0.106	-39.1	0.473				
	Glycerol 2.6% + carbopol 0.05%	96.1	0.085	n.d.	n.d.				
	Glycerol 2.6% + carbopol 0.1%	117	0.144	w.q.	0.478				
	Glycerol 2.6% + carbopol 0.1%	96.0	0.118	n.d.	0.44				
	Glycerol 2.6% + carbopol 0.1%	109.3	0.120	-64.3	n.d.				
	Glycerol 2.6% + carbopol 0.1%	111	0.126	-60.8	0.438				
	Glycerol 2.6% + carbopol 0.1%	101	0.104	w.q.	0.525				
	Glycerol 2.6% + carbopol 0.1%	101	0.138	-33.2	n.d.				
	Glycerol 2.6% + carbopol 0.1%	99.6	0.129	n.d.	n.d.				
	Glycerol 2.6% + carbopol 0.1%	102	0.127	n.d.	n.d.	101	0.138	-30.7	0.516
	Glycerol 2.6% + carbopol 0.1%	n.d.	n.d.	n.d.	n.d.	120	0.156	-43.3	0.488
**c+aNE**	Glycerol 2.6% + carbopol 0.05%	144	0.380	n.d.	n.d.				

n.d., not determined, PC, preconcentrate; PDI, polydispersity index; T, temperature before dilution for measurement; w.q., without quality; Z-Ave, mean hydrodynamic size.

Using glycerol 2.6% as isotonizing agent an adequate viscosity was thereafter obtained for the aNE and osmolality was close to 300 mOsmol/Kg (**Table S6**).

Table S6. Characterization of the osmolality and viscosity of nanoemulsions of segesterone acetate (SA) prepared isotonic carbopol/glycerol aqueous phase (Glycerol 2.6% + Carbopol 0.05%). Nanoemulsions were dilutes to targe Segesterone acetare concentration of 0.48 mg/mL (2.1% PC)

**Code name**	**Osmolality (mOsmol/Kg)**	**Viscosity (mPa·s)**	**Velocity used in the viscosity measurement (rpm)**
aNE^{water} **(dil. in Gly.Carb.0.05%)**	300	111	3
c+aNE **(dil. in Gly.Carb.0.05%)**	294	24	15

PC, preconcentrate

The rheological characterization over time in two different batches of the aNE (=.1% Carbopol) stored at different temperatures is shown in detail (**Fig. S1**).

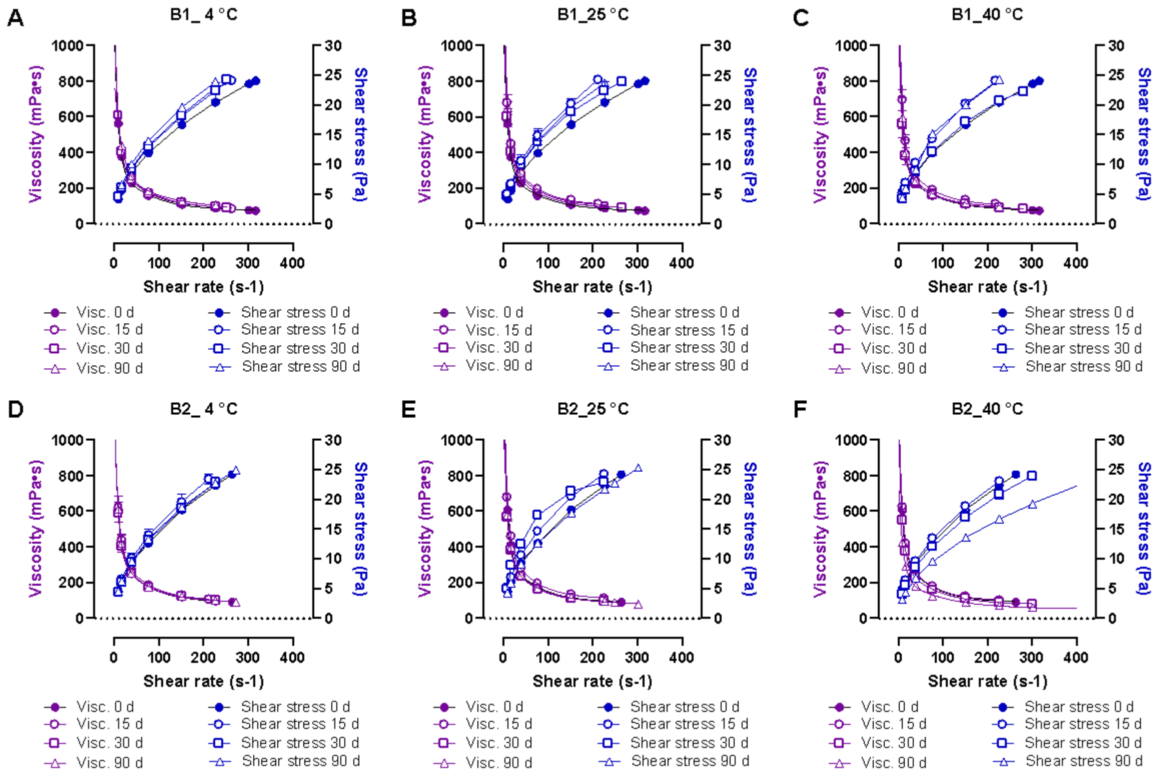

Fig. S1 Detail of the rheological characterization of two batches of the selected anionic and viscous segesterone acetate nanoemulsion (aNE). Viscosity and shear stress at varying shear rates is shown for different time points in 2 batches, up to 90 days, in formulations stored at 4 °C (A and D), 25 °C (B and E) and 40 °C (C and F). All measurements were performed at 25 °C. Each batch was measured twice and data correspond to mean ± standard deviation.

Finally, different batches of the ME had consistently mean droplet sizes around 20 nm and PDI close to or under 0.1, with a few exceptions (**Table S7**). The drug was at times quantified over the target value, therefore excess drug might have, at times, influenced the results. The reduction of the initial concentration of SA in the preconcentrate might increase reproducibility, given the chance for over saturation of the drug might have influenced some of the results.

Table S7 Characterization of the microemulsion (ME) without or with segesterone acetate (SA) prepared in saline at room temperature. Target SA was 0.48 ng/g (target preconcentrate concentration of 2.6%).

Aqueous phase used when diluting the SMEDDS	Without filtration			After filter-sterilization		
	Z-Ave (nm)	PDI	SA assay (mg/mL)	Z-Ave (nm)	PDI	SA assay (mg/mL)
NaCl 0.45%	19.8	0.087	0.985			
NaCl 0.45%	20.9	0.168	0.887			
NaCl 0.45%	22.2	0.104	n.d.			
NaCl 0.9%	19.5	0.079	0.340			
NaCl 0.9%	20.1	0.101	n.d.	19.7	0.102	0.357
NaCl 0.9%	19.4	0.060	n.d.	19.2	0.050	0.493
NaCl 0.9%	20.4	0.149	n.d.			
NaCl 0.9%	21.4	0.097	n.d.			
NaCl 0.9%	25.0	0.218	n.d.			

n.d., not determined, PC, preconcentrate; PDI, polydispersity index; T, temperature before dilution for measurement; w.q., without quality; Z-Ave, mean hydrodynamic

S2. Segesterone acetate HPLC-UV-Vis/DAD assay validation data

As shown in **Fig. S2**, none of the excipients of the SMEDDS after a 100-fold dilution, nor of the neutral NE diluted 1000 times, showed significant interference in the SA retention time (3.1 min), ensuring the selectivity of the present method using the selected chromatographic conditions.

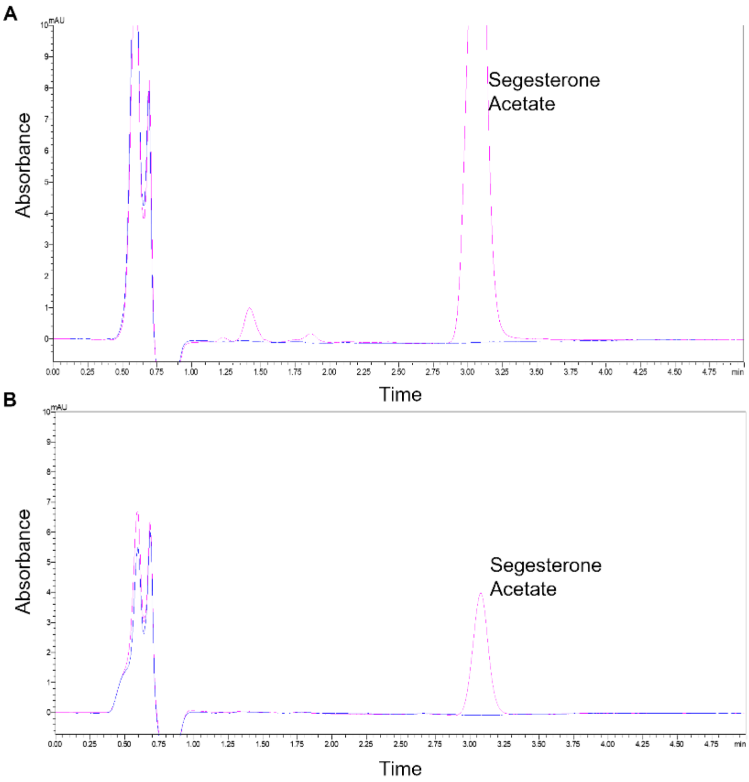

Fig. S2 Representative chromatograms of neutral SMEDDS 100-fold diluted (A) and of neutral NE 1000-fold diluted (B) containing segesterone acetate (pink chromatogram) overlapped with the respective blank formulation (blue chromatogram).

After fitting the SA area of the calibration solutions prepared on three independent days against the nominal concentrations using the weighting factor $1/x^2$, the obtained calibration curve (y = 44723.87795 + 3.16587) exhibited good linearity over the studied calibration range, being it demonstrated by a coefficient of determination near 1 ($r^2 = 0.9969$). The validated method also showed acceptable intraday and interday precision and accuracy, with CV values being less than 8.5%, and bias values being within -6.8% and 6.2% (**Table S8**).

Table S8 Intra and interday precision and accuracy obtained for the quality control samples (QC_{LLOQ}, QC_1, QC_2 and QC_3) for segesterone acetate in the formulations.

Quality control		Intraday (*n* = *5*)			Interday (*n* = *3*)		
	C_{nom} (µg/mL)	C_{exp} (µg/mL)	Precision (% CV)	Accuracy (% bias)	C_{exp} (µg/mL)	Precision (% CV)	Accuracy (% bias)
**QC_{LLOQ}**	0.05	0.051 ± 0.003	6.7	3.0	0.051 ± 0.004	8.5	1.9
**QC_1**	0.149	0.139 ± 0.003	1.9	-6.8	0.146 ± 0.008	5.4	-2.3
**QC_2**	12.454	11.606 ± 0.387	3.3	-6.8	12.054 ± 0.358	3.0	-3.2
**QC_3**	22.417	23.521 ± 0.726	3.1	4.9	23.813 ± 0.804	3.4	6.2

Bias - deviation from nominal value; CV - coefficient of variation;

S3. Characterization of the primary mixed cortical cultures

Materials and methods

Immunocytochemistry

To perform immunocytochemistry, cells were cultured in 24-well multiwell plates containing coverslips previously coated with poly-D-lysine. At the end of the experiment, cells were fixed in 4% paraformaldehyde for 10 min. After fixation, cells were washed with PBS and permeabilized with PBS supplemented with 0.25% Triton X-100 (Sigma-Aldrich, Cat: T9284) for 10 min. Non-specific bindings were reduced by incubating cells in PBS supplemented with 20% FBS and 0.1% Tween (PBS-T) for 1 h. Subsequently, cells were washed again with PBS-T and incubated overnight at 4 °C with the following primary antibodies: mouse anti-MAP2 (1:500, Santa Cruz Biotechnology, sc-74421), rabbit anti-GFAP (1:2000; DAKO; Cat Z0334), and rabbit anti-Iba-1 (1:2000; WAKO; Cat 019-19741). On the next day, cells were washed with PBS-T for 15 min and incubated for 2 h at room temperature with the following secondary antibodies: goat IgG against rabbit IgG conjugated with Alexa 546 (1:1000, Invitrogen, A11010, A546), goat IgG against mouse IgG conjugated with Alexa 488 (1:1000, Invitrogen, A11001, A488). After this procedure, cells were washed again with PBS-T for 15 min and incubated with Hoechst 33342 (1:1000, Invitrogen, H3569) for 10 min. Finally, cells were washed with PBS-T, and coverslips were mounted in Dako mounting medium (Cat: S3023). Images were acquired using an AxioImager Z2 epifluorescence microscope, with an AxioCam camera and Plan-Apochromat 63x/1.4 Oil DIC M27 lenses (WD 0.19 mm). Image acquisition and processing were performed with ZEN-Blue software (ZEN 2.5 PRO).

Cell Counting

To estimate the number of neurons, astrocytes, and microglia present in the culture, the percentage of cells (nuclei marked with Hoechst 33342) that showed labeling for MAP2, GFAP, or Iba1 was determined. Cells from three different cultures were used in this assessment, and three coverslips/experimental conditions were prepared for each culture. Images of 30 fields were acquired on each coverslip.

Results

The percentage of neurons, astrocytes, and microglia present in the primary mixed cortical cultures was estimated by evaluating the staining for MAP2, GFAP, or Iba-1, respectively, by immunocytochemistry (**Fig. S3**). The culture predominantly featured neurons, with approximately 50% of the cells expressing the neuronal marker MAP2. Approximately 5% of the cells in the culture (identified by the nuclear marker Hoechst 33342) expressed GFAP, and 4% of the cells expressed the microglial marker Iba-1. About half of the cells in the culture were not

positive for any of the markers MAP2, Iba-1, or GFAP. It is noted that in culture, there are cells expressing very low amounts of these marker proteins, making it difficult to identify their phenotype.

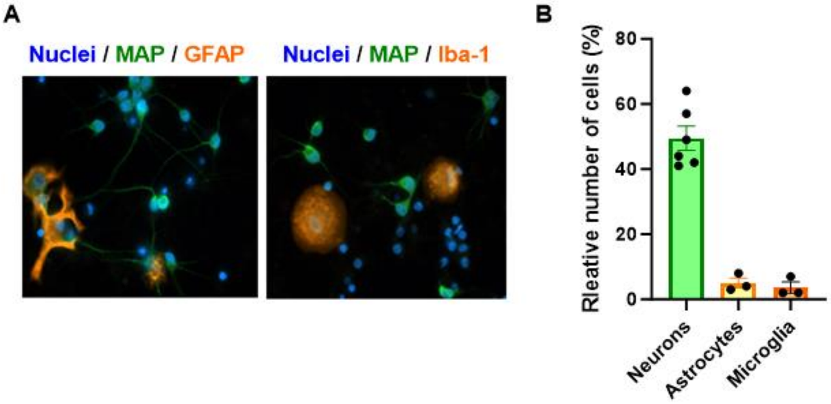

Fig. S3 Characterization of the cellular composition of rat embryonic cortex cultures on the 11th day of culture. A - Representative images of immunostaining of MAP2 (green), GFAP (orange), Iba-1 (orange), Nuclei were labeled with Hoechst 33342 (blue). Images were acquired with a 63x objective. B - Quantification of neurons, astrocytes, and microglia present in the culture, relative to the total number of cells. Results are expressed as a percentage of neurons, astrocytes, and microglia normalized to the total number of cells per field and represent the mean ± SEM of at least three independent cell cultures, with each experimental condition performed in triplicate.

S4. Skin irritation test

Materials and method

The procedure followed the specifications of the Skin irritation in vitro test from OCDE (TG439_EpiDerm).

Tissue Preparation and Pre-incubation

The reconstituted human epiderm (EpiDerm™, EPI-200-SIT, MatTek, Ashland, EUA) was acquired in inserts of 0.63 cm^2. Upon receipt, EpiDerm™ tissues were carefully inspected, cleaned and pre-incubated 60 min as instructed by the manufacturer (37 °C, 5% CO_2, and 90 ± 10% relative humidity), transferred to new wells containing fresh medium (DMEM/low glucose, BioSera), and incubated under the same conditions overnight (18–24 h).

Pre-tests

Several pre-tests (color interference assessment, assessment of direct 3-(4,5-Dimethylthiazol-2-yl)-2,5-diphenyltetrazolium bromide (MTT) reduction, interference with mesh assessment) were performed as specified, and confirmed the non-interference of the formulations with the test.

Test

Tissues were topically exposed to 30 µL of the test sample, as well as 30 µL of positive (5% SDS, Matek) and negative (DPBS, Matek) controls, in triplicates. Given the liquid nature of the test substance, a mesh was applied to ensure uniform spreading across the tissue surface, as recommended by the assay system manufacturer. Following the application, the plates were placed in the incubator for 35 min. Subsequently, the exposure plates

were placed in a biological safety cabinet for 60 min, then rinsed with a saline solution (PBS). Inserts were gently wiped with sterile gauze and cotton swabs to remove excess moisture from the exterior and interior, respectively., and then transferred to new plates for post-incubation. After 24 h ± 2 h, the medium was refreshed, and plates were incubated for an additional 18 h ± 2 h.

MTT Assay

After the 42-hour post-incubation period, tissues were visually inspected and subsequently transferred from the assay medium (EPI-100-NMM, Matek) to wells containing MTT solution (0.3 mL of sterile 1 mg/mL MTT in assay medium per well). Plates were incubated for 3 h ± 5 min at 37 °C and 5% CO_2, protected from light. After incubation, tissues were dried, and the blue formazan salt was extracted using isopropanol. Plates were sealed in plastic bags, wrapped in aluminum foil, and incubated at room temperature for at least 2 h under gentle shaking (~120 rpm). Following extraction, all tissues were pierced to mix the upper and lower extracts. Extracts were homogenized by pipetting, and duplicate optical density (OD) measurements at 570 nm were obtained for each tissue using 200 µL aliquots.

Percentual viability was calculated by normalizing the absorbance values of the test samples to the absorbance of the negative control, after subtraction of the absorbance of wells without tissue (blank). According to the OCDE guideline, above 50% viability the substances are classified as non-irritant.

Results

Cell viability of skin treated with the nNE, even at the highest concentration of 11.2 mg/g (50% of preconcentrate) was not different from the negative control (**Fig. S4**), confirming the safety of the formulations in case of skin contact.

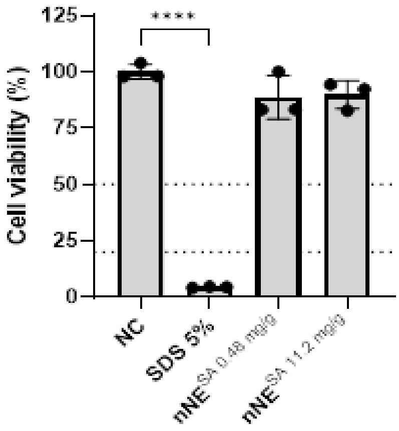

Fig. S4 Skin irritation test result. NC, Sulbecco's phosphate-buffered saline; SDS, sodium dodecyl sulfate; nNE, neutral nanoemulsion; SA, segesterone acetate. Data correspond to individual values as well as mean and standard deviation. **** p < 0.0001, One-way ANOVA with Dunnett's multiple comparisons test.

S5. Bibliography

1. Meirinho S, Rodrigues M, Ferreira CL, Oliveira RC, Fortuna A, Santos AO, et al. Intranasal delivery of lipid-based nanosystems as a promising approach for brain targeting of the new-generation antiepileptic drug perampanel. International Journal of Pharmaceutics. 2022;622:121853.

2. Pires PC, Fernandes M, Nina F, Gama F, Gomes MF, Rodrigues LE, et al. Innovative Aqueous Nanoemulsion Prepared by Phase Inversion Emulsification with Exceptional Homogeneity. Pharmaceutics. 2023;15:1878.

3. Gama F, Meirinho S, Pires PC, Tinoco J, Martins Gaspar MC, Baltazar G, et al. Simvastatin is delivered to the brain by high-strength intranasal cationic SMEDDS and nanoemulsions. Drug Deliv and Transl Res [Internet]. 2025 [cited 2025 Mar 9]; Available from: https://link.springer.com/10.1007/s13346-024-01769-6
